# Breast tumor microbiome regulates anti-tumor immunity and T cell-associated metabolites

**DOI:** 10.1038/s41598-026-48719-5

**Published:** 2026-04-16

**Authors:** Chin-Chih Liu, Dennis Grencewicz, Karthik Chakravarthy, Lin Li, Ruth Liepold, Matthew Wolf, Lynn M. Marcho, Naseer Sangwan, Alice Tzeng, Rebecca Hoyd, Sachin R. Jhawar, Stephen R. Grobmyer, Zahraa Al-Hilli, Andrew P. Sciallis, Daniel Spakowicz, Ying Ni, Charis Eng

**Affiliations:** 1 Genomic Medicine Institute, Lerner Research Institute, Cleveland Clinic, 9500 Euclid Avenue, NE50, Cleveland, OH 44195 USA; 2 The Ohio State University College of Medicine, Columbus, OH 43201 USA; 3 Pelotonia Institute for Immuno-Oncology at The Ohio State University Comprehensive Cancer Center, 460 W12th Ave., BRT 480, Columbus, OH 43220 USA; 4 Center for Immunotherapy and Precision Immuno-Oncology, Cleveland Clinic, Cleveland, OH 44195 USA; 5 Microbiome Composition and Analytics Core, Lerner Research Institute, Cleveland Clinic, Cleveland, OH 44195 USA; 6 Department of Medical Oncology, Dana–Farber Cancer Institute, 450 Brookline Ave., Boston, MA 02215 USA; 7 Department of Radiation Oncology, The Ohio State University Comprehensive Cancer Center, Columbus, OH USA; 8 Cleveland Clinic Abu Dhabi, Oncology Institute, Abu Dhabi, United Arab Emirates; 9 Breast Center, Integrated Surgical Institute, Cleveland Clinic, Cleveland, OH 44195 USA; 10 Department of Anatomic Pathology, Robert J. Tomsich Pathology and Laboratory Medicine Institute, Cleveland Clinic, Cleveland, OH 44195 USA; 11 Department of Molecular Medicine, Cleveland Clinic Lerner College of Medicine, Case Western Reserve University, 9500 Euclid Avenue, NA20, Cleveland, OH 44195 USA; 12 Center for Personalized Genetic Healthcare, Medical Specialties Institute, Cleveland Clinic, Cleveland, OH 44195 USA; 13 Taussig Cancer Institute, Cleveland Clinic, Cleveland, OH 44195 USA; 14 Department of Genetics and Genome Sciences, Case Western Reserve University School of Medicine, Cleveland, OH 44106 USA; 15 Case Comprehensive Cancer Center, Germline High-Risk Cancer Focus Group, Case Western Reserve University, Cleveland, OH 44106 USA

**Keywords:** Microbiome, Tumor-infiltrating lymphocytes, Metabolism, Breast cancer, *Staphylococcus aureus*, Tumor microenvironment, Biomarkers, Cancer, Immunology, Microbiology, Oncology

## Abstract

The breast tumor microbiome has emerged as a potential regulator of tumor immunity, yet its interactions with intratumoral lymphocytes and metabolites remain poorly defined. Here, we investigated relationships among CD8^+^ tumor-infiltrating lymphocytes (TILs), the breast tumor microbiome, and tumor metabolome. In a cohort of 46 breast cancer patients, *Staphylococcus* was the only bacterial genus whose intratumoral abundance positively correlated with cytotoxic CD8^+^ T cell markers and innate-like T cell signatures, including multiple KLR-family receptors. Several metabolites were significantly associated with CD8^+^ TILs, among which NADH, γ-glutamyltryptophan, and γ-glutamylglutamate differed between *Staphylococcus*-positive and *Staphylococcus*-negative tumors. Analysis of an independent cohort of 314 treatment-naïve patients further showed that the association between intratumoral *Staphylococcus*, heightened CD8^+^ T cell activity, and the KLR-associated innate-like T cell program was specific to triple-negative breast cancer (TNBC). In TNBC mouse models, direct intratumoral injection of *Staphylococcus aureus* depleted intratumoral NAD metabolites and suppressed tumor growth by activating CD8^+^ TILs. Together, these findings identify a link between low-biomass intratumoral bacteria and local anti-tumor immunity, and highlight *Staphylococcus* and TIL-associated metabolites as potential biomarkers and therapeutic targets for breast cancer immunotherapy.

**Supplementary Information:**

The online version contains supplementary material available at 10.1038/s41598-026-48719-5.

## Introduction

Breast cancer (BC) is the most prevalent cancer globally among women^1^. Treatment and prognosis hinge substantially on the cancer subtype, which is defined by the presence of the markers estrogen receptor (ER), progesterone receptor (PR), and human epidermal growth factor receptor 2 (HER2)^1^. These proteins play pivotal roles in cancer initiation and progression and represent primary targets for therapeutic interventions. Triple-negative breast cancer (TNBC), characterized by the absence of these markers, is the most aggressive BC subtype. It is predominantly treated with chemotherapy and has a high recurrence rate. In the past decade, immunotherapies, particularly immune checkpoint inhibitors (ICIs), have become a transformative cancer treatment. Among BC subtypes, TNBC has demonstrated the best response to immunotherapy due to its higher immunogenicity, leading to the FDA approval of ICIs for certain TNBC patients^2,3^. However, the relatively low response rate and therapeutic resistance remain major challenges of ICI therapy for TNBC, necessitating further investigation into the regulation of anti-tumor immunity.

CD8^+^ tumor-infiltrating lymphocytes (TILs) are critical effectors of the anti-tumor immune response and immunotherapy targets. The enrichment and activation of CD8^+^ TILs in BC are associated with improved treatment outcomes and prognosis^4–6^. Furthermore, using CD8^+^ TILs in adoptive cell therapy has shown efficacy against various cancers. Consequently, understanding the regulatory mechanisms of CD8^+^ TILs could yield novel therapeutic strategies and biomarkers for BC treatment. TIL activities can be inhibited by molecular products derived from the altered tumor metabolism, such as lactate generated by enhanced aerobic glycolysis (known as the Warburg effect) and oncometabolites (including D-2-hydroxyglutarate, succinate, and fumarate) accumulated by TCA cycle dysregulation^7^. To comprehensively decipher regulation of TILs by tumoral metabolites, it requires systematic profiling of the tumor metabolome alongside TIL analysis.

The function of CD8^+^ TILs is also influenced by the microbial communities present within tumors, referred to as the tumor microbiome. Since the majority of microbes in the human body predominantly reside in the gastrointestinal tract, tumors originating from these tissues can exhibit relatively high microbial biomass^8^. This tumor microbiome can impact cancer phenotypes through the modulation of reactive oxygen species, DNA damage responses, signaling pathways, inflammation, and metabolism^9–13^. For instance, in colorectal cancer, increased levels of intratumoral *Fusobacterium nucleatum* impair TIL function and modulate innate immune cells, indicating substantial influences of the tumor microbiome on anti-tumor immunity^14^. Notably, recent studies have suggested that microbes are also present in various types of tissue and solid tumors previously considered sterile, including glioblastoma, ovarian, bone, and breast tumors, albeit at much lower abundance than gastrointestinal tumors^15–17^. In these tumors with low microbial biomass, the impact of the tumor microbiome remains poorly understood.

Given that the tumor microbiome has shown strong correlations with prognosis and responses to treatment, including immunotherapy in certain cancer types, elucidating the composition and functions of the breast tumor microbiome may provide valuable insights into novel therapeutic strategies and prognostic markers^18–24^. Tzeng et al. showed that breast tumors have lower microbial diversity and reduced abundance of specific bacterial taxa, including *Staphylococcus* and *Streptococcus*, compared to normal breast and tumor-adjacent tissues^17^. Preclinical studies suggested that bacteria within breast tumors, such as *Bacteroides fragilis and Fusobacterium nucleatum*, could contribute to breast cancer development and metastasis^25–27^. Further, clinical evidence indicates that antibiotic use correlates with increased breast cancer risk and worse prognosis, suggesting the potential presence of beneficial microbes that could antagonize the development and progression of breast cancer and/or improve treatment efficacy^28–30^. However, it remains unclear whether there are tumor-suppressive bacteria localized within breast tumors that could activate CD8^+^ TILs and influence immunomodulatory metabolites.

This study examines interactions among CD8^+^ TILs, the tumor microbiome, and the metabolome in human breast tumors within a BC cohort without subtype stratification (Cohort A). We then explore the potential functions of *Staphylococcus* in tumors of specific BC subtypes in an independent, larger BC cohort (Cohort B). Beyond these correlative analyses, we employ murine models of TNBC, including 4T1 and EO771, to validate the functions of tumor-localized bacteria. Our results underscore the significant impact of the breast tumor microbiome on anti-tumor immune responses and CD8^+^ TIL-associated metabolites, which has implications for improving and designing cancer immunotherapy.

## Results

### Patient characteristics of Cohort A

To investigate the interrelationships among CD8^+^ TILs, the tumor microbiome, and metabolome, we collected multidimensional data derived from 46 human breast tumors from 46 breast cancer patients encompassing different BC subtypes (Cohort A). The microbiome composition of all 46 tumors from Cohort A has been profiled by 16S rRNA gene sequencing, and transcript levels of immunity-related genes in 41 tumors from this cohort have been analyzed by Nanostring in our earlier investigation^17^. Using additional tumor tissues from the same cohort, this study further conducted tumor metabolome profiling and immunohistochemistry using anti-CD8 and anti-FoxP3 antibodies. For metabolomic comparisons, an additional 25 non-malignant healthy breast tissues obtained from reduction mammoplasty were included as healthy controls. Detailed demographic and clinicopathologic characteristics of patients is summarized in Table 1.

**Table 1:** Patient characteristics (Cohort A).

Variable	Cancer (n = 46)	Healthy (n = 25)
Age at surgery (years)	59 (33–85)	34 (17–54)
Race		
Caucasian	43 (93.5%)	23 (92.0%)
African American	3 (6.5%)	2 (8%)
TMN stage^#^		
1	28 (70.0%)	
2	8 (20.0%)	
3	4 (10.0%)	
Grade		
1	6 (13.0%)	
2	16 (34.8%)	
3	24 (52.2%)	
ER^+^	37 (80.4%)	
PR^+^	33 (71.1%)	
HER2^+^	6 (13.0%)	
TNBC	7 (15.2%)	
Lymph node status		
Negative	22 (47.8%)	
Positive	24 (52.2%)	
Lymphovascular invasion^#^		
No	24 (54.6%)	
Yes	18 (40.9%)	
Intermediate	1 (2.3%)	
Suspicious	1 (2.3%)	
Histology		
IDC	37 (80.4%)	
ILC	4 (8.7%)	
IDC + ILC	4 (8.7%)	
Others	1 (2.2%)	
Tumor size (cm)	2.7 (0.6–15.5)	

Data are presented as “median (minimum–maximum)” or “number of patients (%)”.

^#^Missing data: TMN stage (n = 6), lymphovascular invasion (n = 2). Percentages are calculated from the total number of patients with known values. ER, estrogen receptor; PR, progesterone receptor; HER2, human epidermal growth factor 2; TNBC, triple-negative breast cancer; IDC, invasive ductal carcinoma; ILC, invasive lobular carcinoma.

### ***Staphylococcus*** correlates with CD8^+^ T cell activation and innate-like T cell signatures in human breast tumors

**Fig. 1:**
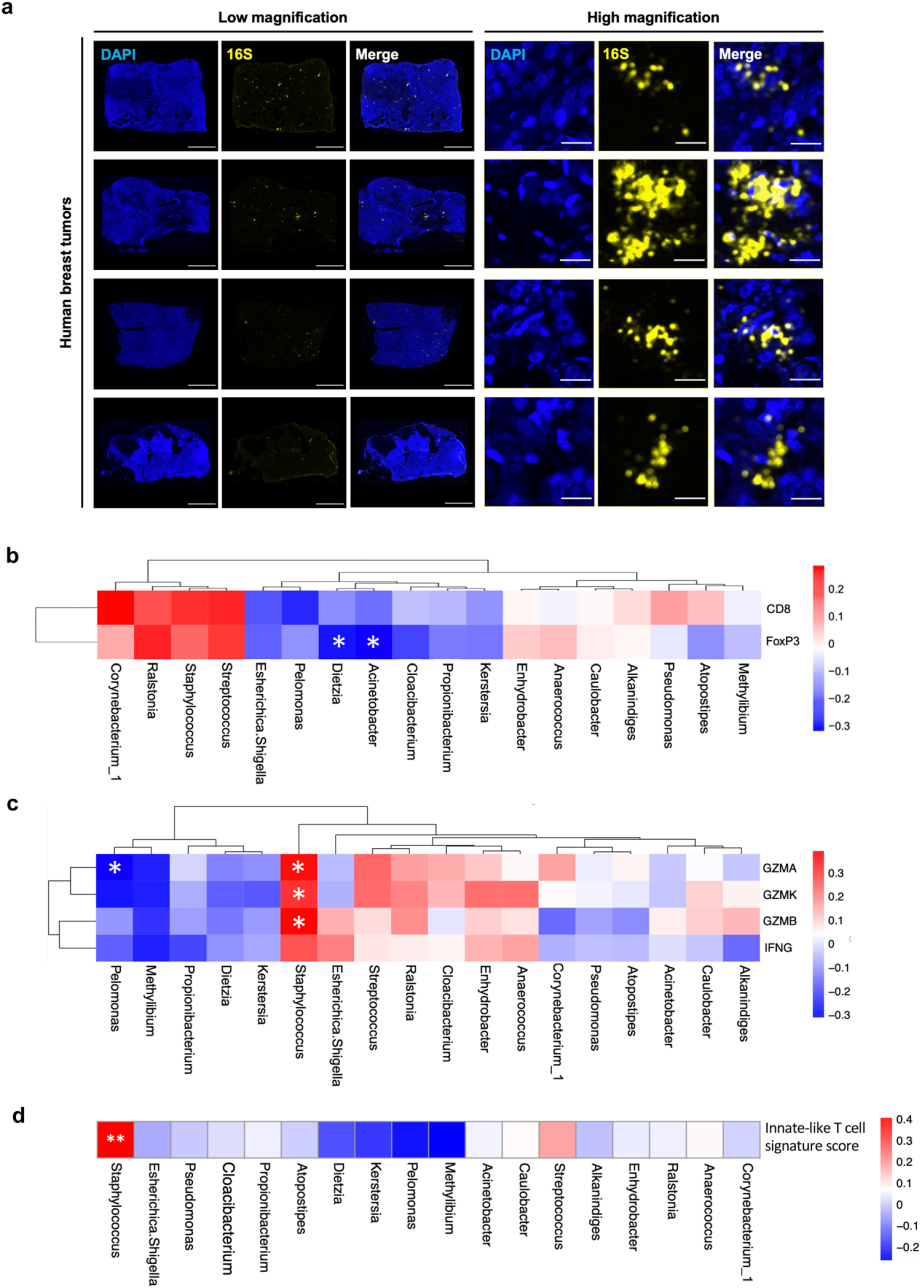
Presence of tumoral bacteria and their correlations with TIL abundance and activation genes. (**a**) RNAscope-fluorescence in situ hybridization (FISH) images of four human breast tumors stained with a probe targeting the bacterial 16S rRNA gene (yellow). Nuclei were counterstained with DAPI (blue). Scale bars, 2000 μm (low magnification) and 25 μm (high magnification). (**b**–**d**) Heatmaps showing Spearman’s correlations between the abundance of bacterial genera and CD8^+^ and FoxP3^+^ cells (**b**), the genes indicative of T cell activation, including GZMA, GZMB, GZMK, and IFNG (**c**), and an innate-like T cell signature score (**d**). Bacterial abundance was assessed using 16S rRNA gene sequencing. *P < 0.05, **P < 0.01.

To independently confirm the presence of bacteria in human breast tumors beyond sequencing-based inference, we performed RNAscope-FISH targeting bacterial 16S rRNA, which revealed discrete punctate signals distributed among tumor cells (Fig. 1a). To determine which bacterial taxa associated with TILs within the breast tumor microbiome, we quantified the relative abundance of 18 dominant bacterial genera identified by 16 s rRNA gene sequencing and assessed CD8^+^ and FoxP3^+^ TIL densities by immunohistochemistry. Transcript levels of genes related to CD8^+^ T cell activation, innate-like T cell markers, and Treg function were extracted from our prior Nanostring profiling^17^.

Correlation analysis showed that among 18 predominant bacterial genera of the tumor microbiome, *Acinetobacter* and *Dietzia* were inversely associated with FoxP3^+^ TIL density, and *Pelomonas* was negatively correlated with GZMA expression (Fig. 1b,c). Strikingly, *Staphylococcus* was the only genus that exhibited statistically significant positive correlations with GZMA, GZMB, and GZMK, and also showed the strongest positive association with IFNG among all taxa (Fig. 1c). *Staphylococcus* was further identified as the only genus significantly associated with a composite innate-like T cell signature, which included multiple KLR family receptor genes (KLRB1, KLRD1, KLRC1, KLRC2, KLRK1), as well as KIR3DL1, TBX21, and FCER1G (Fig. 1d, Supplementary Fig. 1). *Staphylococcus* abundance also displayed a modest, non-significant positive trend with Treg immunosuppressive signature (Supplementary Fig. 2).

To further delineate *Staphylococcus* at the species level, we reanalyzed the 16S rRNA data and identified *S. aureus* as the most abundant *Staphylococcus* species present in Cohort A tumors (supplementary Fig. 3). Taken together, these findings demonstrate that intratumoral *Staphylococcus* is associated with enhanced cytotoxic and innate-like transcriptional programs within TILs, suggesting a potential role for *Staphylococcus*, particularly *S. aureus*, in potentiating local anti-tumor T cell activity in human breast tumors.

### Identification of tumoral metabolites associated with CD8^+^ TILs

To investigate associations between tumoral metabolites and CD8^+^ TILs, we conducted global metabolome profiling of human breast tumors alongside healthy breast tissues as non-malignant controls. Using untargeted metabolomics, we identified over 900 diverse metabolites (supplementary Fig. 4a). As expected, breast tumors were metabolically distinct from healthy breast tissues and exhibited enhanced metabolic activity across various pathways (supplementary Fig. 4b–d). Tumor tissues displayed a higher ratio of glutamate to glutamine, suggesting a higher level of glutaminolysis, consistent with previous reports (supplementary Fig. 4e)^31^. The top 25 metabolites enriched in breast tumors and healthy breast tissues are listed in supplementary Tables 1 and 2, respectively. Breast tumor metabolites correlated with key clinicopathologic features, including cancer stage, histological grade, tumor size, lymph node status, histological subtypes, and patient age (supplementary Fig. 5a–e). Furthermore, the glutamate-to-glutamine ratio varied across tumor stages and cancer subtypes (Supplementary Fig. 6a,b), in agreement with prior studies^31,32^.

**Fig. 2:**
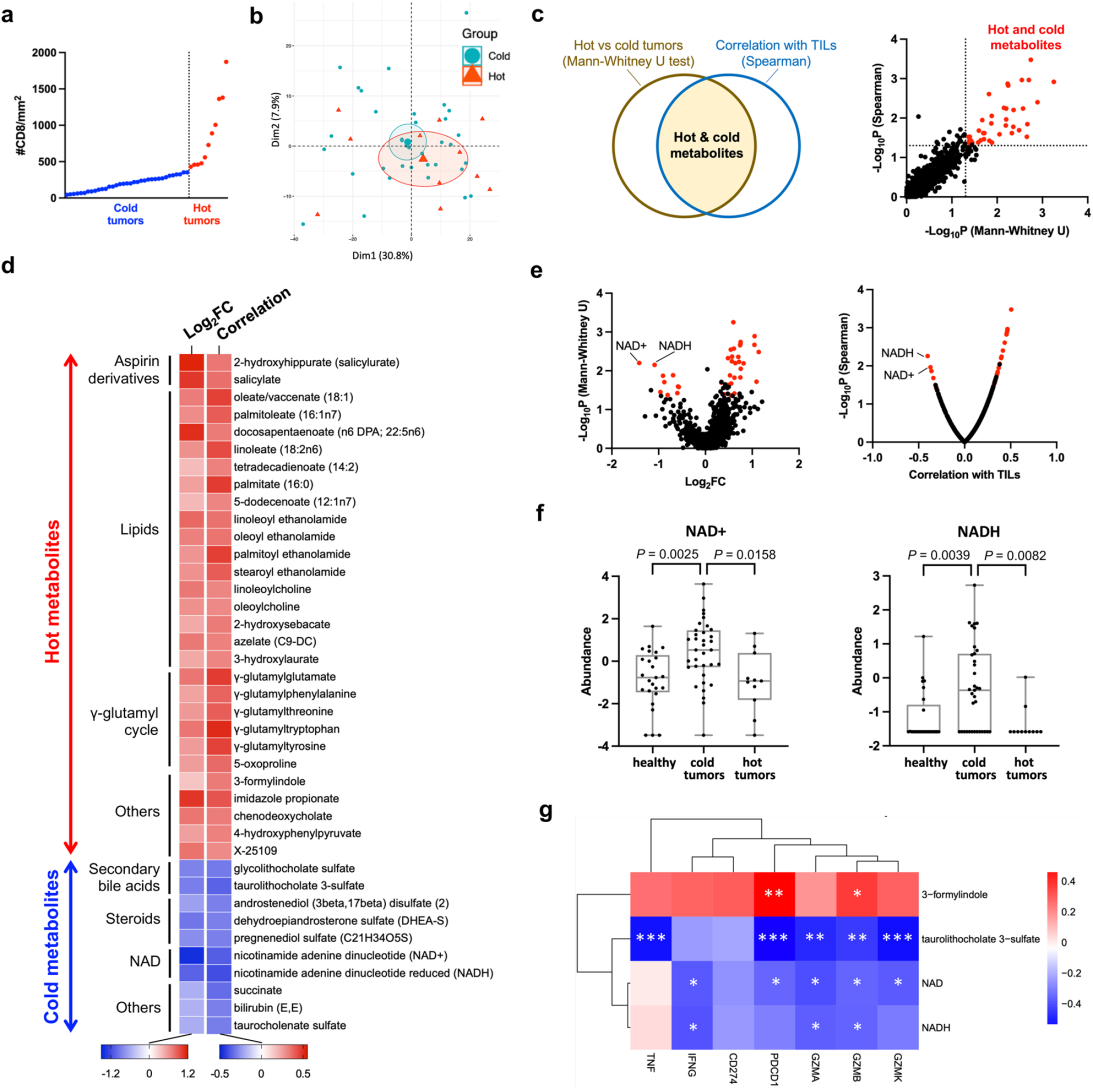
Identification of tumoral metabolites associated with CD8^+^ TILs. (**a**) Categorization of hot and cold tumors based on CD8^+^ cell density quantified using immunohistochemistry. (**b**) Principal component analysis (PCA) of metabolites in hot and cold tumors (n = 11 and 35, respectively). (**c**) The left panel shows the two statistical approaches employed to identify hot and cold metabolites. The right figure depicts hot and cold metabolites that exhibit significant p-values in both analyses in red. (**d**) Heatmap showing the identified hot and cold metabolites. The color gradient represents the log_2_ fold change (FC) of each metabolite between hot and cold tumors (left column) and the correlation between each metabolite and CD8^+^ cell density (right column). (**e**) Volcano plot showing hot and cold metabolites (both highlighted in red) derived from Mann–Whitney U test (left) and Spearman’s rank correlation (right). (**f**) The abundance of NAD^+^ (left panel) and NADH (right panel) in healthy breast tissues (Healthy, n = 25), cold tumors (n = 35), and hot tumors (n = 11). (**g**) Heatmap representing correlations between transcript levels of T cell-related genes and the abundance of NAD^+^, NADH, taurolithocholate 3-sulfate, and 3-formylindole. One-way analysis of variance (ANOVA) with multiple comparisons (**f**). Spearman’s correlation (**g**). *P < 0.05; **P < 0.01; ***P < 0.001.

Breast tumors were stratified into hot and cold groups based on the mean intratumoral CD8⁺ T cell density quantified by immunohistochemistry, corresponding to tumors with relatively higher or lower CD8⁺ T cell infiltration (Fig. 2a). Principal component analysis (PCA) did not reveal global metabolic differences between hot and cold tumors, and the glutamate-to-glutamine ratio remained comparable between the two groups (Fig. 2b, Supplementary Fig. 6c). We then employed two statistical methods to identify metabolites associated with CD8^+^ TILs. The Mann–Whitney U test highlighted metabolites with differential abundance between hot and cold tumors, while Spearman’s rank correlation identified metabolites correlated with CD8^+^ cell density. A total of 39 metabolites exhibited significant p-values in both analyses (Fig. 2c), of which 29 and 10 metabolites were enriched in hot and cold tumors, respectively (henceforth referred to as hot and cold metabolites; Fig. 2d). Hot metabolites encompassed various lipids including medium/long chain fatty acids and N-acylethanolamines (NAEs), compounds of the γ-glutamyl cycle, metabolic aspirin derivatives, and microbiota-derived 3-formylindole and imidazole propionate. Cold metabolites included succinate, bilirubin (E,E), secondary bile acids, nicotinamide adenine dinucleotide (NAD), and steroids including dehydroepiandrosterone sulfate (DHEA-S), androstenediol (3beta,17beta) disulfate, and pregnenediol sulfate (C21H34O5S). Notably, NAD^+^ and NADH emerged as the most significant cold metabolites that exhibited significantly heightened abundance in cold tumors compared to hot tumors and healthy breast tissues (Fig. 2e,f). Additionally, several hot and cold metabolites including NAD^+^, NADH, taurolithocholate 3-sulfate, and 3-formylindole were found to associate with not only the abundance of CD8^+^ cells but also the expression levels of genes related to T cell activation, suggesting their associations with both CD8^+^ TIL abundance and activity (Fig. 2g).

### ***Staphylococcus*** correlates with CD8^+^ TIL-associated tumoral metabolites

**Fig. 3:**
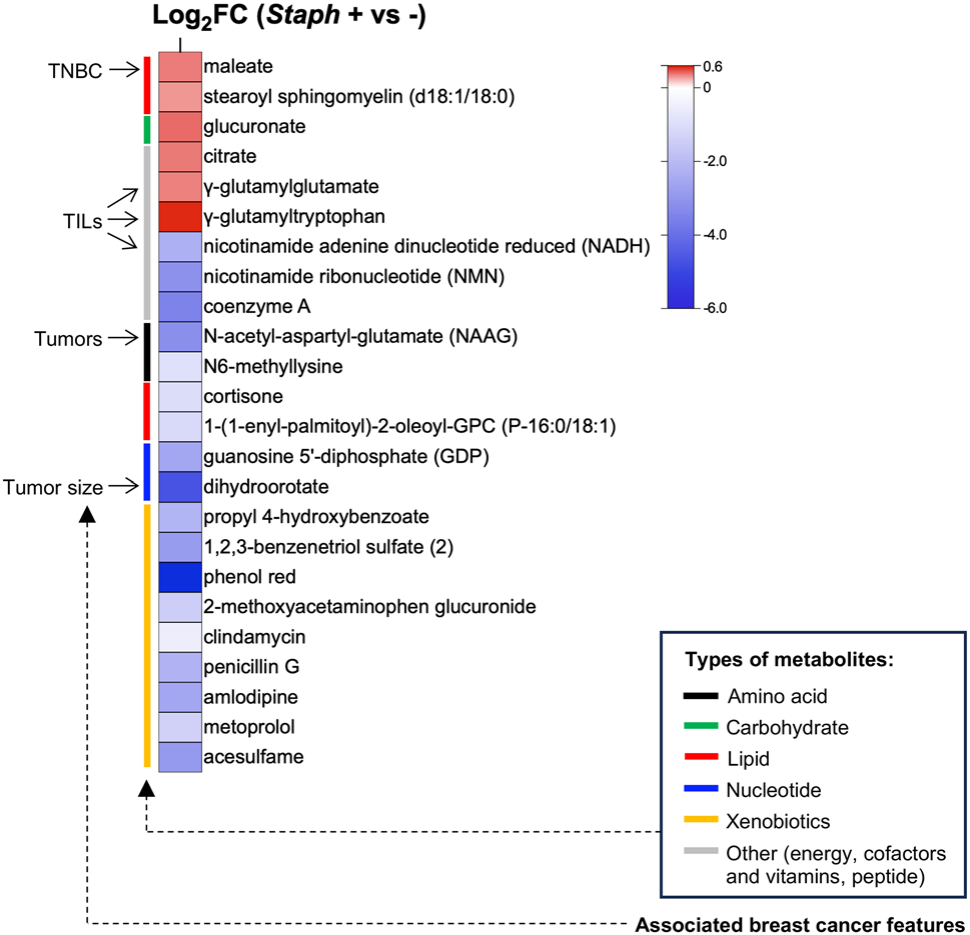
*Staphylococcus-*associated tumoral metabolites. Heatmap displaying differentially abundant metabolites in *Staphylococcus*-positive compared to *Staphylococcus*-negative (*Staph* + vs *-*) human breast tumors. The color gradient represents the log_2_ fold change (FC) relative to the *Staphylococcus*-negative group. Types of metabolites and their associated breast cancer features (detailed in Fig. 2, supplementary Fig. 5, and supplementary Table 1) are indicated on the left side of the heatmap. Unpaired two-tailed Student’s t-test.

Our investigation then turned toward exploring the crosstalk between the tumoral microbiome and metabolites. Sparse canonical correlation analysis (sCCA) revealed that distinct bacterial genera associated with different patterns of metabolites (supplementary Fig. 7). *Anaerococcus* and *Methylibium* were the only genera significantly correlated with the ratio of glutamate to glutamine (supplementary Fig. 8). Since *Staphylococcus* is the only bacterial genus that exhibited significant correlation with T cell activity (Fig. 1c), we conducted a targeted analysis to determine whether *Staphylococcus* associates with the metabolites linked to CD8^+^ TILs and clinicopathological features of breast cancer. Our findings indicated that *Staphylococcus*-positive tumors displayed a decreased level of cold metabolite NADH, along with higher levels of hot metabolites γ-glutamyltryptophan and γ-glutamylglutamate (Fig. 3). Additionally, *Staphylococcus*-positive tumors demonstrated reduced levels of N-acetyl-aspartyl-glutamate (NAAG), dihydroorotate, and GDP as well as increased maleate, of which NAAG, dihydroorotate and maleate are compounds found to be associated with breast tumor, tumor size, and TNBC, respectively (supplementary Fig. 5, and supplementary Table 1). These results reveal specific associations between *Staphylococcus* and CD8^+^ TILs-associated metabolites in breast tumors.

### ***Staphylococcus*** is associated with enhanced CD8^+^ T cell cytotoxicity and activation of KLR-associated innate-like T cell programs in TNBC

**Fig. 4:**
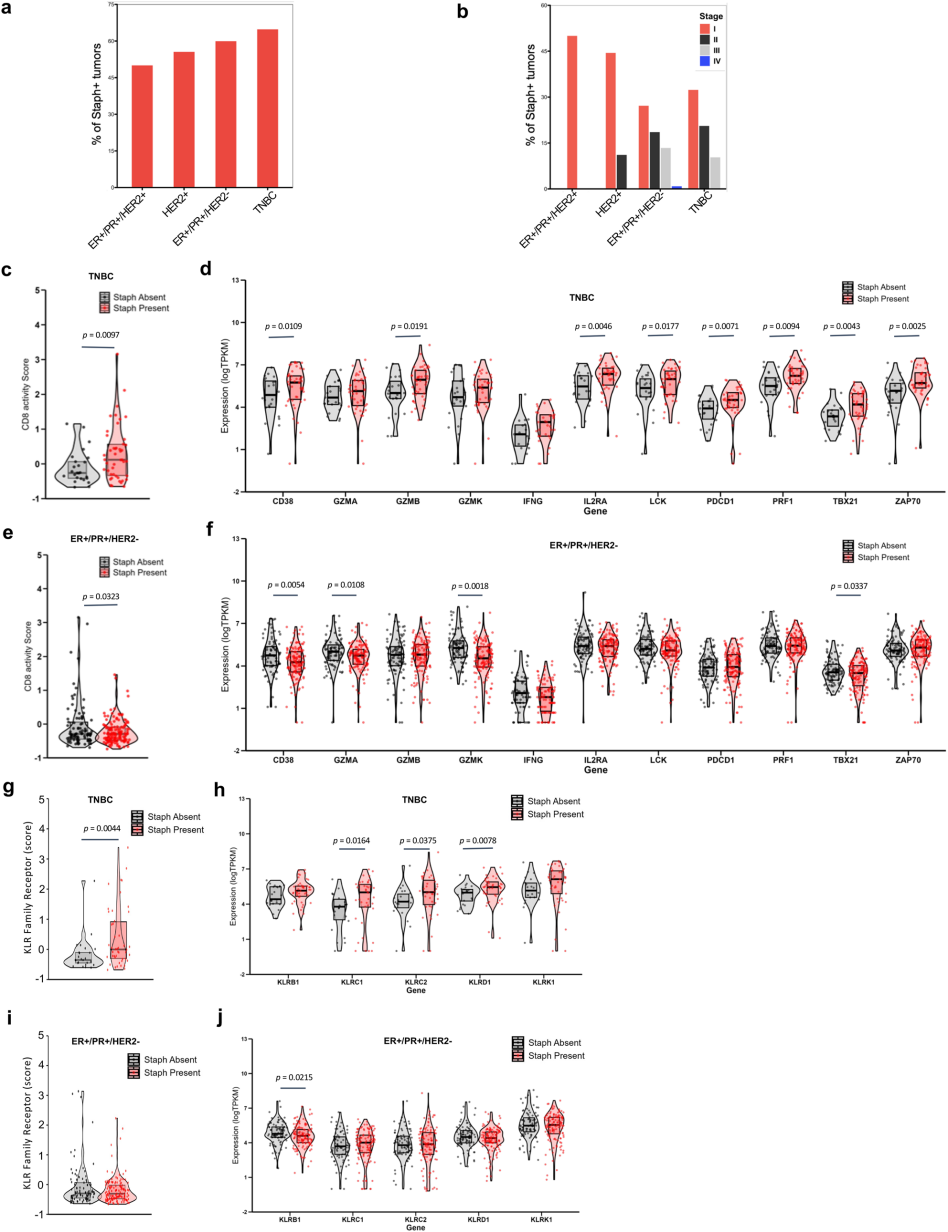
Associations between *Staphylococcus*, clinicopathological features, and CD8^+^ TILs. (**a**, **b**) Percentages of Cohort B human breast tumor samples identified as positive for *Staphylococcus* (Staph + tumors) across cancer subtypes (**a**) and stages (**b**). (**c**–**f**) Comparisons of CD8^+^ T cell activity score and related gene expression in TNBC (**c**, **d**) and ER + /PR + /HER2^-^ tumors (**e**, **f**) with and without the presence of *Staphylococcus* (shown as red and black dots, respectively). (**g–j**) Comparisons of KLR family receptor score and related gene expression in TNBC (**g**, **h**) and ER + /PR + /HER2- tumors (**i**, **j**). Z-score transformed signature scores and log-transformed transcripts per kilobase-million (TPKM) counts were compared by t-test. Only statistically significant differences are shown with p-values.

To evaluate whether intratumoral *Staphylococcus* is associated with immune variation across breast cancer subtypes, we examined RNA-seq data from an independent cohort of 314 treatment-naïve breast tumors (Cohort B; Table 2). The proportion of *Staphylococcus*-positive tumors was comparable across major intrinsic breast cancer subtypes (Fig. 4a) and exhibited a consistent, though nonsignificant, decreasing trend with advancing stage within each subtype (Fig. 4b). *Staphylococcus* presence was not significantly associated with overall prognosis, nor with outcomes within either TNBC or ER^+^/PR^+^/HER2^-^ subgroups (Supplementary Fig. 9).

**Table 2:** Patient characteristics (Cohort B).

Variable	Cancer (n = 314)
Age at diagnosis (years)	54 (46–63)
Race	
White	290 (92.4%)
African American or black Ko	19 (6.1%)
Asian	2 (0.6%)
Unidentified	3 (1.0%)
STAGE	
1	122 (38.9%)
2	117 (37.2%)
3	65 (20.7%)
4	7 (2.2%)
Unidentified	3 (1.0%)
ER^+^/PR^+^/ HER2^-^	232 (73.9%)
ER^+^/PR^+^/HER2^+^	2 (0.6%)
HER2^+^	9 (2.9%)
TNBC	71 (22.6%)

Data are presented as “median (IQR)” or “number of patients (%)”.

Percentages are calculated from the total number of patients (n = 314). ER, estrogen receptor; PR, progesterone receptor; HER2, human epidermal growth factor 2; TNBC, triple-negative breast cancer.

In TNBC tumors, the overall abundance of CD8^+^ T cells did not differ between *Staphylococcus*-positive and -negative tumors (Supplementary Fig. 10a,b), However, *Staphylococcus*-positive TNBC tumors exhibited significantly higher CD8⁺ T cell activation scores, characterized by elevated expression of GZMB, PRF1, IL2RA, CD38, PDCD1, LCK, ZAP70, and TBX21, with similar nonsignificant upward trends in GZMA, GZMK, and IFNG (Fig. 4c,d). In contrast, *Staphylococcus*-positive ER^+^/PR^+^/HER2^-^ tumors displayed lower CD8⁺ T cell activation scores and reduced expression of GZMA, GZMK, CD38, and TBX21 (Fig. 4e,f). Consistent with this pattern, *Staphylococcus*-positive TNBC tumors showed higher Treg-associated immunosuppressive activity, along with increased expression of PDCD1, LAG3, ENTPD1, and IL2RA (Supplementary Fig. 11). In contrast, this association between *Staphylococcus* and Treg-related immunosuppressive activity was not observed in ER^+^/PR^+^/HER2^−^ tumors.

We next evaluated innate-like T cell programs. In TNBC, *Staphylococcus*-positive tumors exhibited significant enrichment of the KLR family receptor signature (KLRB1, KLRD1, KLRC1, KLRC2, KLRK1; p = 0.0044), whereas signatures corresponding to innate/NK-like cytotoxic marker (NKG7), inhibitory and activating KIR receptors (KIR2DL1, KIR2DL3, KIR3DL1, and KIR2DS4), and innate-like T cell lineage markers (FCER1G and TBX21) showed no significant differences (Fig. 4g,h, Supplementary Fig. 12a–l). In ER^+^/PR^+^/HER2^−^ tumors, KLR signatures were similar regardless of *Staphylococcus* status (Fig. 4i,j). These results indicate a selective enhancement of KLR-associated innate-like programs in *Staphylococcus*-positive TNBC, rather than a generalized activation of all NK- or NKT- related pathways.

When modeled as a continuous variable, *Staphylococcus* abundance showed significant correlations in TNBC with reduced M0 macrophages and increased monocytes, and a near-significant association with activated CD4 memory T cells (p = 0.0859; Supplementary Fig. 13, Additional file 1). Beyond these significant results, TNBC tumors exhibited a distinct correlation profile not seen in ER^+^/PR^+^/HER2^-^ tumors or in the combined cohort, in which higher *Staphylococcus* abundance aligned with relatively increased M1 macrophages and activated NK cells and decreased M2 macrophages and resting NK cells. Although these additional correlations did not reach statistical significance (adjusted p-values > 0.4), the TNBC-restricted pattern is consistent with a shift toward a more pro-inflammatory immune environment associated with *Staphylococcus* in TNBC.

Finally, *Staphylococcus* presence was not associated with alterations in host metabolic pathways (NAD metabolism or γ-glutamyl cycle), suggesting a potential direct modulation of T cell–associated metabolites (Supplementary Fig. 14). Collectively, these results indicate that tumor-associated *Staphylococcus* is linked to enhanced CD8⁺ T cell cytotoxicity and selective activation of KLR-associated innate-like T cell programs specifically within TNBC.

### Intratumoral *S.aureus* enhances CD8⁺ T cell–dependent anti-tumor immunity and depletes NAD metabolites in TNBC models

**Fig. 5:**
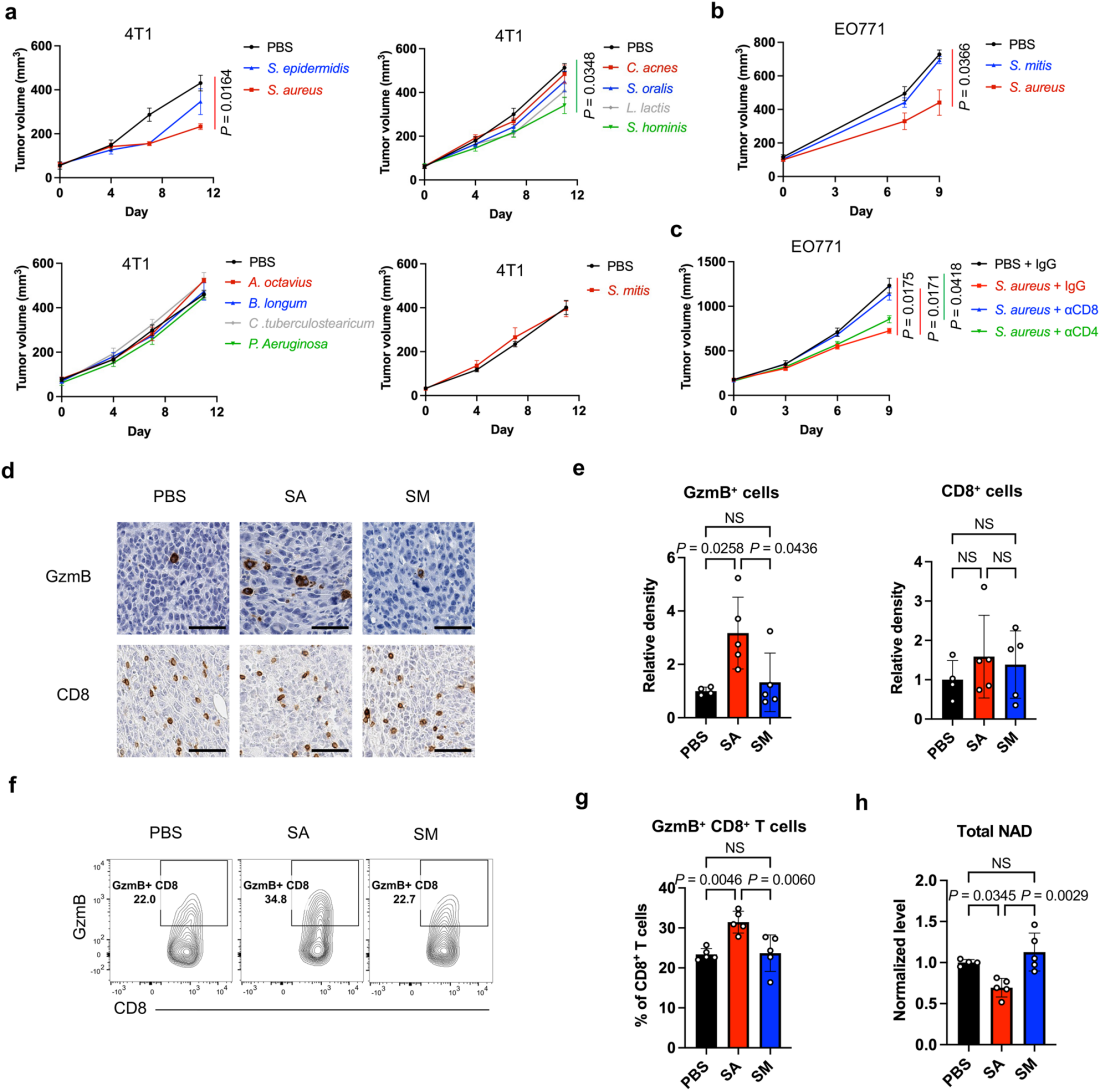
Intratumoral *S. aureus* modulates CD8^+^ TILs and total NAD level in TNBC models. (**a**, **b**) Growth of 4T1 (**a**) and EO771 (**b**) tumors after intratumoral injection of various bacterial species compared to PBS-treated control (n = 3–5). (**c**) Growth of EO771 tumors after intratumoral injection of *S. aureus* with and without antibody-based depletion of CD8^+^ or CD4^+^ T cells (n = 4). (**d**) Representative immunohistochemistry (IHC) images showing GzmB^+^ and CD8^+^ cells in EO771 tumors after intratumoral injection of *S. aureus* (SA) or *S. mitis* (SM), with PBS treatment as a control. Scale bars are shown in black at 50 μm. (**e**) Quantification of densities of GzmB^+^ and CD8^+^ cells based on IHC staining shown in (**d**). (**f**) Representative contour plots of flow cytometric analysis showing the percentage of GzmB-expressing CD8^+^ T cells in EO771 tumors after intratumoral injection of *S. aureus* or *S. mitis*, with PBS treatment as a control. (**g**) Quantification of the percentage of GzmB^+^ cells among CD8^+^ T cells based on the flow cytometric plots shown in (**f**). (**h**) Total levels of NAD^+^ and NADH in the EO771 tumors 10 days after intratumoral injection of *S. aureus* or *S. mitis*, with PBS treatment as a control. Two-way analysis of variance (ANOVA) with multiple comparisons (**a**–**c**). One-way ANOVA with multiple comparisons (**e**, **g**, **h**). NS, not significant.

To functionally evaluate whether *Staphylococcus* and other tumor-associated bacteria modulate TIL activity and anti-tumor immunity, we examined multiple bacterial species previously detected in human breast tumors based on prior 16S rRNA sequencing data, including *Staphylococcus aureus*, *Staphylococcus epidermidis*, *Staphylococcus hominis*, *Streptococcus mitis*, *Streptococcus oralis*, *Cutibacterium acnes*, *Lactococcus lactis*, *Anaerococcus octavius*, *Bifidobacterium longum*, and *Corynebacterium tuberculostearicum*. Data from the 4T1 preclinical model of triple-negative breast cancer (TNBC) showed that, in most cases, intratumoral bacteria as modelled by intratumoral bacterial injections did not influence tumor growth (Fig. 5a). In contrast, *S. aureus* and S. hominis significantly suppressed tumor growth, with *S. aureus* producing the strongest effect. The tumor-suppressive activity of *S. aureus* was independently validated in the EO771 TNBC model using PBS and *S. mitis* as negative controls (Fig. 5b; Supplementary Fig. 15)^33^.

Mechanistic studies demonstrated that *S. aureus*–mediated tumor inhibition was dependent on CD8⁺ T cells, as depletion of CD8⁺ cells—but not CD4⁺ cells—abrogated the anti-tumor effect (Fig. 5c). Immunohistochemistry confirmed that *S. aureus* significantly increased intratumoral GzmB⁺ cells without altering overall CD8⁺ T cell numbers (Fig. 5d–e). Flow cytometry further showed robust induction of GzmB in CD8⁺ T cells following *S. aureus* treatment in both EO771 and 4T1 models (Fig. 5f,g; Supplementary Fig. 16), whereas *S. epidermidis* and *S. mitis* had no effect on GzmB expression or tumor growth (Fig. 5a,b; Supplementary Figs. 15, 16, 17). In the EO771 model, intratumoral *S. aureus* also increased IFNγ and TNFα expression in CD4⁺ T cells and reduced Treg abundance (Supplementary Figs. 18, 19a, 20a).

Beyond lymphocytes, *S. aureus* remodeled the tumor myeloid compartment. Although dendritic cells were unaffected, *S. aureus* reduced tumor-associated macrophages, decreased the M2/M1 ratio, and lowered monocytic MDSCs (mMDSCs) while increasing granulocytic MDSCs (gMDSCs) and polymorphonuclear leukocytes (PMNs) in the EO771 model (Supplementary Figs. 19b–f, 20b–f). *S. aureus* also appeared to attenuate the immunosuppressive potential of mMDSCs, gMDSCs, and PMNs by reducing phosphorylated signal transducer and activator of transcription 3 (STAT3) (supplementary Fig. 19g,h)^34^. Notably, the ability of *S. aureus* to enhance CD8^+^ T cell activity and modulate myeloid populations in EO771 tumors was largely recapitulated in 4T1 tumors. Similar alterations were observed in macrophages, mMDSC, and gMDSCs and PMNs (supplementary Fig. 21), with the exception that Treg levels remain unchanged in 4T1.

Given the strong correlation between *Staphylococcus* abundance and the cold metabolite NADH in human breast tumors (Fig. 3), we next evaluated whether *S. aureus* directly influences total NAD level. Indeed, intratumoral *S. aureus* but not *S. mitis* significantly reduced total NAD levels in EO771 tumors (Fig. 5h, supplementary Fig. 22). Collectively, these findings demonstrate that the presence of *S. aureus* in preclinical TNBC tumors depletes the cold metabolite NAD and activates CD8^+^ TILs, thereby enhancing anti-tumor immune responses. These functional data align with the positive associations observed between *Staphylococcus* abundance and T cell activity in human breast tumors. (Figs. 1c, 4c,d).

## Discussion

The influence of the breast tumor microbiome on tumorigenesis, immune regulation, and disease progression remains poorly characterized, in part due to the extremely low microbial biomass in breast tissues and the difficulty of establishing causality in human tumors. By integrating two independent clinical cohorts with preclinical TNBC models, this study provides convergent evidence that *Staphylococcus*—particularly *S. aureus*—modulates local anti-tumor immunity and tumor metabolism.

In Cohort A, *Staphylococcus* was the only bacterial genus positively correlated with cytotoxic T cell markers and innate-like T cell signatures. Because of sample size limitations, subtype-stratified analyses were not feasible in this cohort. Analysis of a larger cohort (Cohort B) revealed that the association between *Staphylococcus*, heightened CD8⁺ T cell activity, the KLR-associated innate-like T cell programs, and increased Treg-associated immunosuppressive activity was found to be restricted to TNBC, suggesting a subtype-specific interaction between the tumor microbiome and the immune microenvironment. Furthermore, *Staphylococcus*-positive TNBCs exhibited elevated PDCD1 expression, a biomarker linked to responsiveness to anti–PD-1 therapy. These findings raise the possibility that *Staphylococcus* may modulate TNBC sensitivity to anti–PD-1 immunotherapy, a hypothesis that warrants further investigation.

Innate-like T cells, such as KLR-expressing cytotoxic T cells, MAIT cells, NKT cells, and other unconventional subsets, provide rapid barrier-associated immune responses and are increasingly recognized as contributors to breast tissue homeostasis and tumor immunity^35–41^. Across both cohorts, *Staphylococcus* abundance was consistently associated with selective upregulation of KLR family receptor genes, whereas other innate-like module genes (KIR receptors, NKG7, TBX21, FCER1G) showed no consistent differences. This pattern suggests that intratumoral *Staphylococcus* preferentially enhances KLR-driven innate-like cytotoxic circuits rather than globally activating all NK- or NKT-related pathways, raising the possibility that specific microbial cues tune discrete arms of the unconventional T cell compartment.

The immune remodeling associated with *Staphylococcus* in human TNBC tumors from Cohort B was recapitulated in the 4T1 and EO771 mouse models following intratumoral administration of *S. aureus*. In Cohort B, *Staphylococcus*-positive TNBC tumors exhibited elevated CD8 activity scores, and when analyzed as a continuous variable, higher *Staphylococcus* abundance was associated with fewer undifferentiated macrophages, increased monocyte abundance, and a trend toward enhanced CD4⁺ memory T cell activation. *Staphylococcus* abundance in TNBC tumors also displayed signs of macrophage and NK cell polarization toward pro-inflammatory phenotypes, a pattern not observed in ER⁺/PR⁺/HER2⁻ tumors, supporting a subtype-specific association between *Staphylococcus* and immune remodeling. Consistent with these human correlations, intratumoral *S. aureus* administration in both mouse models enhanced CD8⁺ T cell cytotoxicity (GzmB⁺), reduced tumor-associated macrophages and monocytic MDSCs, and increased granulocytic MDSCs and polymorphonuclear leukocytes. In EO771 tumors, *S. aureus* further decreased the M2/M1 macrophage ratio, mirroring the shift from M2- to M1-like macrophages observed in *Staphylococcus*-high human TNBCs. *S. aureus*-treated EO771 tumors also exhibited increased IFNγ⁺ and TNFα⁺ CD4⁺ T cells, reinforcing the positive association between *Staphylococcus* abundance and activated CD4^+^ memory T cells in TNBC. Collectively, these findings indicate that *Staphylococcus* drives TNBC-specific, pro-inflammatory immune remodeling.

Our data also showed that Intratumoral *S. aureus* suppressed tumor growth in a CD8-dependent manner, directly linking the observed immune remodeling to tumor control. This intervention was also accompanied by a reduction in intratumoral NAD metabolites, consistent with the metabolic correlations identified in human tumors. These data suggest that *S. aureus* may enhance anti-tumor immunity not only by selectively activating KLR-driven innate-like T cell programs but also by modulating the local metabolic environment to favor cytotoxic lymphocyte activity. Together, these clinical and preclinical datasets provide mutually reinforcing evidence that intratumoral *Staphylococcus* shapes the TNBC microenvironment by enhancing CD8⁺ T cell cytotoxicity, selectively activating innate-like KLR programs, and remodeling both myeloid populations and tumor metabolism, highlighting potential mechanistic pathways for microbiome-mediated anti-tumor immunity.

Interestingly, in human breast tissues, the abundance of *Staphylococcus* consistently showed a decreasing trend with advancing cancer stages across various breast cancer subtypes (Fig. 4b). Furthermore, the abundance of *Staphylococcus* was lower in breast tumors than in healthy breast tissues^17^. These observations raise the hypothesis that *Staphylococcus* species may function as “sentinel bacteria” within breast tissue, supporting anti-tumor immunity during early tumor development. Similar tumor-suppressive activities of *S. aureus* have also been observed in glioblastoma, where the presence of intracranial *S. aureus* in several patients were associated with longer survival^42^. Whether the presence of *Staphylococcus* influences responses to immunotherapy, and whether similar or distinct functions occur in ER⁺/PR⁺/HER2⁻ tumors, warrant further investigation.

In addition to the tumor microbiome, deregulated cancer metabolism impairs TIL activities through the abnormal accumulation or depletion of immunomodulatory metabolites. This study systematically investigated the tumoral metabolites associated with CD8^+^ TILs using untargeted metabolome profiling and TIL analysis. Notably, our findings recaptured several metabolites previously identified to have immunomodulating functions in cancer. For example, among the hot metabolites, i.e., metabolites that are enriched in tumors with dense CD8^+^ TILs, linoleate and the microbiota-derived 3-formylindole (also called indole-3-aldehyde) were reported to stimulate cytotoxic T cell (CTL)-dependent anti-tumor immunity (Fig. 2d)^43,44^. Hot metabolites also include salicylate and 2-hydroxyhippurate (salicylurate), both of which are metabolic derivatives of aspirin (acetylsalicylic acid). Interestingly, aspirin intake has been found to be associated with better responses to programmed death 1 (PD-1)/programmed death ligand 1(PD-L1)-based immunotherapy^45,46^. As for cold metabolites, we showed that NAD^+^ and NADH are the most significant compounds that negatively correlate with both TIL abundance and T cell cytokines. Consistent with our findings, a previous study indicated that supplementation with NAD^+^ inhibited CD8^+^ TIL function^47^. The other cold metabolites, taurolithocholate 3-sulfate and glycolithocholate sulfate, are derivatives of secondary bile acids, whose functions in antagonizing CTL-mediated tumor cell killing were recently identified in colorectal cancer^48^. The cold metabolites also include sex hormone precursors DHEA-S and androstenediol (3beta,17beta) disulfate (2), which could be converted to testosterone and/or estrogen. Their desulfated forms DHEA and androstenediol (3beta,17beta) also retain weak estrogenic activity. Accordingly, estrogen signaling may be linked to the suppression of TIL function. While succinate was identified as a cold metabolite, other TCA cycle intermediates did not exhibit significant association with TILs, suggesting succinate-mediated TIL regulation might be independent of the alterations of TCA flux. In addition to these characterized metabolites, our study uncovered many TIL-associated compounds with unreported immunomodulatory functions, which are potential agents or targets for immunotherapy that require further validation.

In this study, we reported potential interactions between microbes and tumoral metabolites. For instance, the abundance of *Anaerococcus* was positively associated with an increased ratio of glutamate to glutamine, a potential indicator of glutaminolysis that is associated with breast cancer progression and TNBC (supplementary Figs. 4, 6)^31,32^. It is possible that enhanced tumoral glutaminolysis fosters an environment suitable for colonization by anaerobic *Anaerococcus* and other anaerobic bacteria, since the increased glutaminolysis could lead to hypoxia through oxidative phosphorylation-mediated oxygen consumption^49^. We also found that *Staphylococcus*-positive tumors exhibit reduced levels of GDP and the cold metabolite NADH, as well as increased abundance of hot metabolites including γ-glutamyltryptophan and γ-glutamylglutamate. It is likely that tumor-localized *Staphylococcus* directly consumes NADH and GDP to synthesize NADP(H) and alarmones (p)ppGpp, respectively, conferring survival advantage inside host cells similar to activity in other harsh conditions^50,51^. Furthermore, we demonstrated in a preclinical BC model that intratumoral administration of *S. aureus*, but not *S. mitis*, led to a reduction of total NAD level in tumors, demonstrating that *Staphylococcus* plays a causal role in altering NAD and NADH.

While the precise means by which *S. aureus* activates CD8^+^ TILs in preclinical TNBC models remain unclear, we posit several potential mechanisms may be involved. *S. aureus* could modulate the CD8^+^ TIL-associated metabolites such as NAD and NADH and secrete various immunomodulatory agents, such as α-hemolysin, superantigens, and extracellular vesicles. In particular, α-hemolysin has been reported to stimulate CD8^+^ TILs and impede TNBC tumor growth^52^. Additionally, peptides from *S. aureus* can be presented on the surface of tumor cells in cancer patients, which has been demonstrated with activity to elicit CTLs^53^. Furthermore, *S. aureus* invasion could stimulate the STING and RIG-I-like receptor signaling pathways by inducing DNA damage^54,55^. Future studies are needed to validate these and other crosstalk mechanisms.

Our study has several limitations that warrant consideration. First, the small sample size of Cohort A constrained the statistical power of our analyses. As a result, initial assessments of statistical significance in Cohort A relied on unadjusted p-values without correction for multiple testing. To mitigate this limitation, we implemented a multi-faceted statistical strategy in Cohort A to enhance the reliability of identified TILs-associated metabolites. Additionally, a larger, independent breast cancer cohort (Cohort B) was analyzed to further explore the *Staphylococcus*–TIL relationship across diverse breast cancer contexts. Nevertheless, preliminary findings from Cohort A require further validation in independent datasets. Second, we were unable to culture patient tumor-derived *S. aureus* strains for functional studies due to limited tissue availability, as samples had already been allocated for microbiome, metabolome, and TIL analyses. Third, the mechanisms by which bacteria migrate into human breast tumors remain unclear, and intratumoral injection in murine models may not fully recapitulate the human condition. However, these experiments demonstrated that, among the various breast tumor microbiome species, *S.aureus* uniquely modulates tumor growth and T cell activation, consistent with clinical observations. This suggests that the presence of *S. aureus* within tumors, irrespective of its route of entry, could influence TILs and cancer phenotype. Another constraint is that while our metabolome profiling detected over 900 intratumoral compounds with high sensitivity, it did not capture dynamic flux or cell-type specificity. Future studies could integrate isotope-labeling and multi-omics approaches, including single-cell RNA sequencing, high-plex RNAscope-FISH, high-plex immunofluorescence, spatial transcriptomics, spatial metabolomics, and tumor culturomics. Finally, although *S. aureus* depleted NAD in EO771 tumors, its effect across other models remains to be tested to assess generalizability.

In conclusion, this study identifies microbial taxa and metabolites within human breast tumors that are associated with CD8^+^ TILs. We also explore crosstalk between the tumor microbiome and metabolome, and demonstrate that the presence of *Staphylococcus* within tumors stimulates CD8^+^ TIL-dependent anti-tumor immunity in TNBC and modulates T cell-associated metabolites. These findings illuminate the potential significant roles of the low-biomass tumor microbiome in anti-tumor immunity and cancer metabolism, and lay the groundwork for further studies assessing whether these CD8^+^ TIL-associated microbial taxa and metabolites could serve as biomarkers or therapeutic agents for immunotherapy, increasing its efficacy in TNBC or expanding its usage in other BC subtypes.

## Methods

### Patient enrollment and tissue collection

Cohort A included 46 breast cancer patients and 25 control patients without breast cancer. These patients were drawn from the larger cohort described in our previous publication^17^. For the present study, we selected cases based on the availability of: (i) clearly annotated breast cancer subtype information (ER, PR, and HER2 status); (ii) remaining fresh-frozen tissue suitable for untargeted metabolomic profiling; and (iii) accessible FFPE blocks for CD8⁺ and FoxP3⁺ immunohistochemistry. Tissue samples were obtained as described previously from three biorepositories, including the Cleveland Clinic Breast Center Microbiome Biorepository, Cooperative Human Tissue Network, and Case Comprehensive Cancer Center Human Tissue Procurement Facility^17^. Specimens were obtained using standard biorepository protocols from female patients who provided written informed consent. Breast tumor samples were obtained from oncologic surgery performed at Cleveland Clinic (n = 16), University Hospitals Cleveland Medical Center (n = 9), and The Ohio State University (n = 21). Non-malignant breast tissues were collected from reduction mammoplasty performed at University Hospitals Cleveland Medical Center (n = 10), Hospital of the University of Pennsylvania (n = 7), University of Virginia Medical Center (n = 7), and The Ohio State University (n = 1). An additional file shows detailed demographic and clinicopathologic characteristics of all cancer patients comprising Cohort A (see Additional file 2). To control for possible contamination from environmental microbes, a specimen container filled with 5 ml of sterile saline or water was left open in the operating room during breast surgery at each institution from which specimens were collected (Cleveland Clinic, Hospital of the University of Pennsylvania, The Ohio State University Wexner Medical Center, University Hospitals Cleve- land Medical Center, University of Virginia Medical Center). These environmental controls were also stored at − 80 °C and processed in parallel with tissue specimens^17^.

A pathologist verified that tissue samples from patients without breast cancer (healthy controls) to be free of malignant cells. Histopathological data were compiled from pathology reports. Breast cancer staging was standardized using the American Joint Committee on Cancer/Union for International Cancer Control 8th edition TNM pathologic stage criteria. Specimens were flash-frozen and stored at − 80 °C until further processing. This study was approved by the Cleveland Clinic institutional review board (IRB #14-774 and 17-791).

For Cohort B, 314 BC patients enrolled in a prospective cohort study (IRB protocol 2013H0199) at Ohio State University. Surgical specimens or tissue biopsies were sterilely collected, frozen in liquid nitrogen, and stored at − 80 °C until further processing. Processed clinical and expression data were shared with researchers under an Ohio State University IRB-approved honest broker protocol (2015H0185). Due to the absence of patient metadata on pregnancy history, parity, and breastfeeding status in both Cohort A and B, these variables could not be included in the analysis.

### RNAseq data generation and processing

Fresh-frozen tissue collected from a cohort of 314 breast cancer patients (Cohort B) was subjected to research use only (RUO) grade RNA sequencing at HudsonAlpha (Huntsville, AL) or Fulgent Genetics (Temple City, CA). Qiagen RNAeasy plus mini kit was used to isolate nucleic acid and fragmented to an average insert size of 216 bp. Exons were enriched using the Illumina TruSeq RNA Exome with single library hybridization, followed by cDNA synthesis, library preparation, and sequencing to a depth of 50 M paired-end reads. Adapters were trimmed via k-mer matching, followed by quality trimming and filtering, contaminant filtering, sequence masking, GC filtering, length filtering, and entropy filtering. Cleaned reads were processed for gene expression, microbe identification, and contaminant filtering using exotic v2.1^56^. Briefly, this tool conservatively identifies microbial reads within host-dominated expression datasets. Cleaned and filtered reads are aligned to the telomere to telomere human reference genome. Gene expression signatures were calculated using tnesig^57^. Immune cell abundances were estimated with CIBERSORTx^58^. Tumors were classified as “*Staphylococcus*-positive” if they had greater than 0 microbial reads aligning to the *Staphylococcus* genus in the RNA-seq data. Tumors with zero detected reads were labeled as “*Staphylococcus*-negative”.

### Immune signature score calculation

To assess the activity of key immune programs, including CD8⁺ T cell cytotoxicity, Treg-associated immunosuppressive pathways, and innate-like T cell signatures, we calculated immune signature scores for both Cohorts A and B. For each sample, the expression values of all genes comprising a given signature were first Z-score normalized across the dataset. The immune signature score was then calculated as the average of these Z-scores, providing a summary measure of the signature’s overall activity. Individual genes within each signature were analyzed using normalized expression values: for Cohort A, normalized Nanostring counts were used, whereas for Cohort B, log-transformed transcripts per kilobase-million (logTPKM) values were used.

### RNAscope-fluorescence in situ hybridization (FISH)

All RNAscope-FISH was manually performed with the RNAscope Multiplex Fluorescent Reagent Kit v2 from Advanced Cell Diagnostics (ACD, Newark, CA). In brief, slides of human breast tumor tissues were baked in a 60 °C oven for 2 h and then deparaffinized in accordance with ACD recommendations. Target retrieval was performed with RNAscope Target Retrieval Reagents (PN 322000; ACD) warmed to 99 °C in an Oster steamer for 15 min. Tissue was then subject to protease digestion with Protease Plus (PN 322331; ACD) and placed in a HybEZ II oven (PN 321710; ACD) for 30 min at 40 °C. The protease was rinsed off with DI water. EB-16 s-rRNA-C2 probe (464461-C2; ACD) was applied to the tissue and hybridized in a HybEZII oven for 2 h at 40 °C. Slides were then rinsed with RNAscope Wash Buffer (PN 310091; ACD). Amps 1–3 and Channel 1 from the Multiplex Fluorescent Detection Kit v2 (PN 323110; ACD) were applied to tissue in sequence and incubated in a HybEZII oven at 40 °C for the duration recommended by ACD. The probe was visualized with Opal 570 (FP1488001KT; Akoya Biosciences) diluted in RNAscope Multiplex TSA Buffer (322809; ACD). Tissue was then stained with RNAscope Multiplex FL v2 DAPI (323108; ACD) and coverslipped using ProLong Gold antifade aqueous mounting media (P36930; Invitrogen). Images were acquired at 40X magnification on the Vectra Polaris Quantitative Pathology Imaging System.

### Immunohistochemistry, image analysis, and categorization of hot and cold tumors

The formalin-fixed, paraffin-embedded human breast tumor tissues were sectioned at 5 μm. The CD8^+^ and FoxP3^+^ cells present in these human tissues were identified by immunohistochemistry double stain using a DISCOVERY ULTRA automated stainer (Roche) followed by analysis using CaloPix, a computational pathology diagnostic software powered by artificial intelligence (Tribun Health, Paris). Details of these procedures were described in our previous study with a modification where CD8 was visualized by a DISCOVERY Yellow detection kit (Roche #760-228) after being stained with the primary and secondary antibodies^17^. CaloPix after trained by our images was used to identify CD8^+^ and FOXP3^+^ cells within tumor tissues. Identification of tumor-infiltrating CD8^+^ and FOXP3^+^ cells by CaloPix was validated by a breast pathologist. The densities of CD8⁺ and FOXP3⁺ cells in tumors from Cohort A are available in an additional file (see Additional file 3). The cut-off between hot and cold tumors in Cohort A was defined by the mean CD8⁺ TIL density (356.03 cells/mm2); tumors with densities above this mean were classified as hot, and those below as cold.

Mouse tumor samples were prepared and subjected to immunohistochemistry based on the procedure described in our previous study with modifications as follows^52^. The primary antibody used for CD8 (Cell Signaling, #98941, 1:50) was diluted in a blocking solution and incubated at 4 °C overnight. The primary antibody for granzyme B (Abcam, #ab4059, 1:1500) was diluted in a blocking solution and incubated at room temperature for 1 h. After being washed with PBS (Dulbecco’s phosphate-buffered saline), tumor sections were incubated with horseradish peroxidase (HRP)-conjugated secondary antibody at room temperature for 30 min. The sections were subsequently stained with 3,3′-Diaminobenzidine (DAB) Substrate Kit and hematoxylin. Images were captured at 40X magnification on a Leica Aperio AT2 Slide Scanner and analyzed with QuPath digital pathology software. For the enumeration of stained cells, the function “Detect Positive Staining” was used to determine the positively stained pixels with DAB optical density (OD) value higher than 0.2 and 0.4 in the staining of granzyme B and CD8, respectively.

### *Staphylococcus* species profiling from 16S rRNA sequencing data

To characterize the *Staphylococcus* composition in tumor samples, we re-examined the amplicon sequence variants (ASVs) generated in our prior 16S rRNA gene sequencing workflow, as previously described^17,59,60^. ASVs annotated to the genus *Staphylococcus* in the DADA2-generated feature table were extracted for downstream analysis. Species-level assignments were based on the taxonomic annotations returned by the original classifier. For each sample, the relative proportions of *Staphylococcus* species shown in Supplementary Fig. 3 reflect the fraction of ASV reads attributed to each species out of all reads assigned to *Staphylococcus* at the species level.

### Measurement of global untargeted metabolites

Untargeted metabolomics measurements were conducted at Metabolon (Morrisville, NC, USA) using Ultrahigh Performance Liquid Chromatography-Tandem Mass Spectroscopy (UPLC-MS/MS). Proteins were precipitated and removed from samples with methanol under vigorous shaking. The resulting extract was divided into five aliquots, dried, and reconstituted in solvents compatible with each of the methods containing a series of standards at fixed concentrations to ensure injection and chromatographic consistency. Among the five aliquots, two were analyzed by two separate reverse phase (RP)/UPLC-MS/MS methods with positive ion mode electrospray ionization (ESI), of which one was chromatographically optimized for more hydrophilic compounds while the other one was chromatographically optimized for more hydrophobic compounds. Two aliquots were analyzed by RP/UPLC-MS/MS and hydrophilic interaction liquid chromatography (HILIC)/UPLC-MS/MS, respectively, with negative ion mode ESI. One aliquot was reserved for backup. This strategy ensured maximal recovery and coverage of metabolites. All methods utilized a Waters ACQUITY ultra-performance liquid chromatography (UPLC) and a Thermo Scientific Q-Exactive high-resolution/accurate mass spectrometer interfaced with a heated electrospray ionization (HESI-II) source and Orbitrap mass analyzer operated at 35,000 mass resolution. Raw data was extracted, peak-identified and QC processed using Metabolon’s hardware and software. Compounds were identified by comparison to the Metabolon-maintained library based on the retention index, accurate mass match to the library + /- 10 ppm, and the MS/MS score based on a comparison of the ions present in the experimental spectrum to the ions present in the library spectrum. The following QC and curation processes ensure accurate and consistent identification of true chemical entities and remove those representing system artifacts, misassignments, and background noise.

The raw values of the abundance of each metabolite were acquired according to the integrated area under the curve, which were then divided by the median of those samples to give each metabolite a median of one. The minimum value of each metabolite in the median scaled data is imputed for the missing values. Then the data were divided by the mass of input tissues and re-scaled to have a median equal to one. Finally, the metabolome data were transformed using the natural log function, which was used for statistical analysis and can be found in an additional file (see Additional file 4).

### Canonical correlation analysis

We employed a sparse canonical correlation analysis (sCCA) to identify the potential crosstalk between tumoral microbiome and metabolites^61^. Canonical correlation analysis (CCA) has previously been suggested as a potential approach for performing integration analysis^62^. CCA is a technique used to identify weighted linear combinations of features from two distinct data modalities that exhibit significant association. This method aims to uncover relationships between variables measured on the same subjects. The typical CCA model provides non-zero weights to all features, potentially leading to overfitting when dealing with high-dimensional data. The problem of overfitting can be mitigated by incorporating a sparsity penalty into the CCA model, so enabling the inclusion of feature selection^62^.

The sCCA model can be mathematically represented as uTXTZv, subject to the following constraints: The vectors u and v are constrained to have norms not exceeding 1, represented as ‖‖u‖‖2 ≤ 1, ‖‖v‖‖2 ≤ 1. The vectors u and v are then constrained to have zero values for indices outside of specified sets Q1 and Q2, respectively, P1(u) ≤ c1, P2(v) ≤ c2, and uj = 0 for all j ∉ Q1, and vj = 0 for all j ∉ Q2. The paired multi-omics datasets are represented by X and Z. The canonical vectors, u and v, include the weights assigned to each feature. The weighted linear combinations of features within each subject, Xu and Zv, are considered as the canonical scores. The P1 and P2 functions reflect the lasso penalty functions applied to the canonical variates. As a result, the values of u and v become sparse when the values of c1 and c2 are sufficiently tiny. The subsets of features in X and Z that exhibit significant univariate correlation with the phenotype are represented by Q1 and Q2. Conversely, features that do not strongly associate with the phenotype are assigned weights of zero automatically. The model’s optimal tuning parameters were determined using 100 times of permutation.

We conducted sCCA using the PMA package in R to select a parsimonious linear combination of variables that maximizes the correlation between two multivariate datasets^61^. The first dataset comprised normalized and scaled tumoral microbiome, and the second comprised metabolomics measurements from matched samples. Regularization parameters for the sCCA analysis were chosen by a grid search consisting of 100 possibilities of L1 values ranging from 0 (representing the highest sparsity) to 1 (representing the lowest sparsity), with increments of 0.1. We chose the set of L1 values that resulted in the highest canonical correlation of the first variate for further examination.

### Cell culture

The sources, culture media, sterility, and authentication of 4T1 and EO771 cell lines were described in our previous study^52^.

### Bacterial culture

To acquire live suspensions of bacteria, *S. aureus* (BEI Resources Repository, # HM162), *S. epidermidis* (BEI Resources Repository, # HM118), *S. hominis* (ATCC, # 27844), *S. oralis* (ATCC, # 700233), *S. mitis* (BEI Resources Repository, # HM262), *C. acnes* (ATCC, # 6919), and *B. Longum* (ATCC, # 15707) were inoculated into BHI media (Sigma, # 110493) supplemented with menadione (1 mg/l, MP Biomedicals, # 02102259), hematin (1.2 mg/l, Santa Cruz, # sc-207729), histidine (0.2 mM, Tokyo Chemical Industry, # H0149), and L-cysteine hydrochloride (0.5 g/l, J.T.Baker, # 2071-05) and incubated at 37 °C in an anaerobic chamber. *L. lactis* (ATCC, # 19435) was grown aerobically at 37 °C in the aforementioned media supplemented with 5% serum. *C. tuberculostearicum* (ATCC, # 35692) was grown aerobically at 37 °C in PYG media supplemented with menadione (0.315 mg/l), hematin (5 mg/l), histidine (0.834 mM), and L-cysteine hydrochloride (0.5 g/l). On the day of intratumoral injection, bacteria were pelleted down by centrifugation, washed, and resuspended in PBS at a concentration of 5 × 10^9^ CFU (colony-forming units) per ml, which was confirmed by enumeration of plated colonies.

### Tumor growth and treatment

4T1 and EO771 cells were trypsinized and resuspended in PBS without calcium and magnesium. 1 × 10^4^ of 4T1 cells and 2.5 × 10^5^ to 1 × 10^6^ of EO771 cells were injected into the fourth mammary fat pad of six to eight week old female BALB/c and C57BL/6 J mice (The Jackson Laboratory), respectively. The number of injected cells was the same between different treatment groups of every experiment. After palpable tumors formed, mice were randomized with matching tumor volumes into case and control groups before treatment. Tumor volumes were measured by digital calipers and calculated using the ellipsoid formula (L x W x W/2). The day of the first bacterial injection was defined as day zero hereafter. To investigate the effects of intratumoral bacteria on the growth, tumor microenvironment, and total NAD level of EO771 tumors, 2 × 10^7^ CFU of bacteria were injected into palpable tumors on day zero and the tumors were isolated on day 10 for downstream analyses. To characterize the effects of intratumoral bacteria on 4T1 tumor growth, 1 × 10^8^ CFU of bacteria were injected into 4T1 tumors on days zero and day four. To determine the influence of intratumoral bacteria on TILs in 4T1 tumors, 2 × 10^7^ CFU of bacteria were injected into tumors on day zero, and tumors were isolated for immune profiling on day 12. To test whether lymphocytes are required for the *S. aureus*-mediated inhibition of tumor growth, tumor-bearing mice were subjected to intraperitoneal injections of anti-CD4 (BioXCell, clone GK1.5, 150 μg), anti-CD8 (BioXCell, clone YTS 169.4, 150 μg), or isotype IgG control (BioXCell, clone LTF-2, 150 μg) one day before the intratumoral injection of 2 X 10^7^ CFU of *S. aureus*, which was followed by an additional two injections of the same neutralizing antibodies on days three and six.

At the experimental endpoint, mice were euthanized using a Quietek CO₂ system for CO₂ asphyxiation, followed by cervical dislocation to ensure death, in accordance with AVMA guidelines. All animal procedures were approved by the Cleveland Clinic Institutional Animal Care and Use Committee (protocol #00002429) and conducted in compliance with the approved IACUC protocol. Animal experiments were performed and reported in accordance with Animal Research: Reporting of In Vivo Experiments (ARRIVE) guidelines.

### Immune cell dissociation and flow cytometry

Tumors were digested as described previously^52^. For the intracellular analysis of granzyme B, dissociated cells were additionally cultured for 4 h with 50 nM phorbol 12-myristate 13-acetate (PMA, Sigma, # P1585), 500 nM ionomycin (Sigma, # I0634), and Brefeldin A (Biolegend, # 420601) before staining. Collected cells were stained with viability dye (Biotium, # 32018, 1:1000) for 30 min, blocked with anti-mouse CD16/32 antibody (Biolegend, # 156604, 1:100) in staining buffer (PBS with 0.5% Bovine Serum Albumin (BSA), 2 mM EDTA, and 0.05% NaN_3_) for 20 min, and then stained with antibodies targeting cell surface markers for 30 min on ice. After two washes with staining buffer, cells were resuspended in fixation buffer at room temperature for 60 min and permeabilized using the permeabilization buffer according to the manufacturer’s instructions (Biolegend, # 424401; or Thermo Fisher Scientific, # 88-8824-00 for granzyme B analysis). Cells were then stained for 45 min at room temperature with antibodies targeting intracellular markers. Samples were analyzed using a SONY ID7000™ spectral cell analyzer. The resulting FCS data files were then analyzed by FlowJo™ Software v10.8.0 (BD Life Sciences) with the gating strategy shown in Supplementary Fig. 23.

Fluorescence-minus-one (FMO) plus isotype controls were used for intracellular staining of GzmB, IFNγ, and TNFα (Supplementary Fig. 23a,b). For Treg gating, we used in vitro–stimulated splenocytes as a reference. Splenocytes activated with anti-CD3 (2 µg/ml; Bio X Cell, 2C11) and anti-CD28 (1 µg/ml; Bio X Cell, PV-1) served as positive controls for Foxp3 expression, providing gate boundaries for Tregs. Representative reference plots are shown in Supplementary Fig. 23d,e. For other surface markers, gates were defined based on (i) clear separation between positive and negative populations in the fluorescence intensity distribution and (ii) comparison with unstained controls. This gating strategy was applied consistently across all samples within each experiment.

The following antibodies were used in this study: CD45 (BD Biosciences, clone 30-F11), CD11b (Thermo Fisher Scientific or BD Biosciences, clone M1/70), Ly-6C (Biolegend, clone HK1.4), Ly-6G (BD Biosciences, clone 1A8), I-A/I-E (BD Biosciences, clone M5/114.15.2), CD3 (Biolegend, clone 17A2), CD11c (BD Biosciences, clone N418), CD206 (Biolegend, clone C068C2), CD4 (BD Biosciences, clone GK1.5), F4/80 (Biolegend, clone BM8), CD8a (Biolegend, clone 53–6.7), phosphorylated STAT3 (Thermo Fisher Scientific, clone LUVNKLA), Granzyme B (Thermo Fisher Scientific, clone NGZB), IFNγ (Biolegend, clone XMG1.2), TNFα (Biolegend, clone MP6-XT22), FoxP3 (Biolegend, clone MF-14).

### NAD measurement

At 6 or 10 days after bacterial injection, EO771 tumors were isolated, washed with ice-cold PBS, and cut into small pieces using sterile dissection tools inside a positive flow cell culture hood. The total level of NAD^+^ and NADH was measured by the NAD/NADH Quantification Kit (Sigma, # MAK037). In brief, 15 to 30 mg of tumor pieces were homogenized with 700 μl of NADH/NAD Extraction Buffer in a Dounce homogenizer on ice. Following vigorous vortexing and centrifugation, the supernatant was filtered through a 10 kDa cut-off spin filter (Abcam # ab93349) to remove potential NAD^+^/NADH-consuming enzymes. After the addition of NAD Cycling Enzyme and NADH Developer, the absorbance at 450 nm was measured using a microplate reader (BioTek Synergy H1). The protein concentration of the samples before filtration was determined by a bicinchoninic acid (BCA) protein assay (ThermoFisher, # 23227) for the normalization of the results.

### Data normalization and statistical analyses

The microbiome composition data and expression levels of 579 immunity-related genes were obtained from our previous study, which analyzed 46 and 41 human breast tumors from Cohort A, respectively^17^. Only the bacterial genera that were identified in more than 5% of breast tissue samples were included for downstream analysis. The raw values of the abundance of each bacterial genera were normalized to the mass of input tissues used for 16S rRNA sequencing. The further normalization was performed based on the formula $log10(RCn\times \sum xN+1)$, where RC represents the number of counts i for a sample, n is the number of sequences in a sample, the sum of x is the total number of counts in the table, and N is the total number of samples^63^. The immune gene expression data of tumors comprising Cohort A can be found in an additional file (see Additional file 5).

Statistical analyses were performed with R version 4.3.1 and Prism version 10.1.1 (GraphPad). Unpaired two-tailed Welch’s t-test or Mann–Whitney U test was used to compare experiments with two groups. One-way analysis of variance (ANOVA) was used to compare experiments with greater than two groups. To compare tumor growth, Two-way ANOVA with Sidak’s and Tukey’s multiple comparison tests were performed with two and greater than two groups, respectively. *P* < 0.05 was used as a threshold of statistical significance. Spearman’s correlation was applied to analyze the associations between microbiome and TILs, microbiome and T cell genes, metabolites and TILs, and metabolites and T cell genes. Sparse canonical correlation analysis was used to identify the tumoral metabolites associated with specific tumor microbes. Due to the small sample size of Cohort A, most statistical significance for analyses in this Cohort was determined using unadjusted p-values without correction for multiple testing. For the identification of hot and cold metabolites, both Spearman’s correlation and the Mann–Whitney U test were applied in parallel to mitigate type I error. Only metabolites meeting the significance threshold (unadjusted p < 0.05) in both statistical approaches were classified as hot or cold metabolites. Further details are provided in the Results section.

## Supplementary Information

Supplementary Information 1.

Supplementary Information 2.

Supplementary Information 3.

Supplementary Information 4.

Supplementary Information 5.

Supplementary Information 6.

## Data Availability

As previously mentioned, the 16S rRNA gene sequencing data generated in our earlier study have been deposited in the European Nucleotide Archive (ENA) under study accession number PRJEB43655 (https://www.ebi.ac.uk/ena/browser/view/PRJEB43655)^17^. All other data generated or analyzed during this study are included in this published article, its supplementary information files, and its additional files. Custom code scripts are available in the code repository https://github.com/spakowiczlab/staph-brca.
