# Supplementary material for: Breast tumor microbiome regulates anti-tumor immunity and T cell-associated metabolites
Source: Sci Rep. 2026 Apr 16;16:17587. doi: 10.1038/s41598-026-48719-5 (PMC13243561; doi:10.1038/s41598-026-48719-5)
Supplement: Supplementary file 6 — Supplementary Information 6. [file 41598_2026_48719_MOESM6_ESM.pdf]

Supplementary information

Breast tumor microbiome regulates anti-tumor immunity and T cell-associated metabolites

Supplementary figures and legends 1 to 23

Supplementary tables 1 to 2

Supplementary figure 1

a

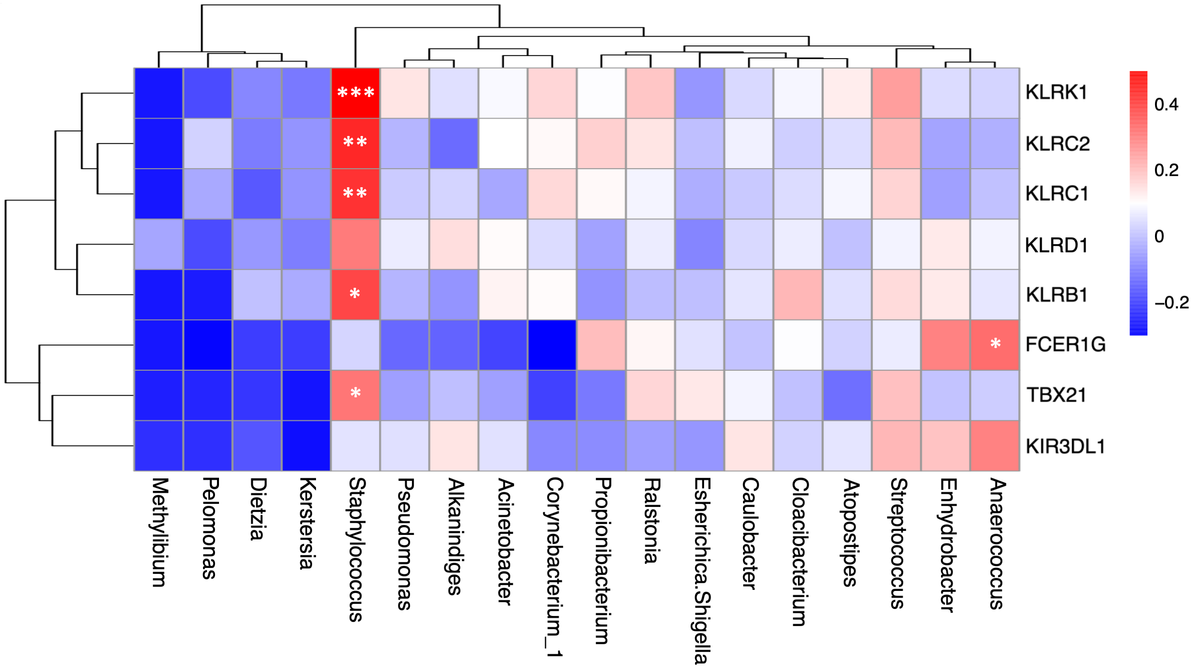

Supplementary figure 1 | Correlations between intratumoral bacterial genera and individual genes comprising the innate-like T cell signature. Heatmap showing Spearman correlations between the abundance of 18 bacterial genera and the eight genes used to define the innate-like T cell transcriptional signature (KLRB1, KLRD1, KLRC1, KLRC2, KLRK1, TBX21, FCER1G, KIR3DL1) in Cohort A. Individual gene-level correlations are presented to complement the composite signature correlation shown in Fig. 1d. *, $P < 0.05$; **, $P < 0.01$; ***, $P < 0.001$.

Supplementary figure 2

a

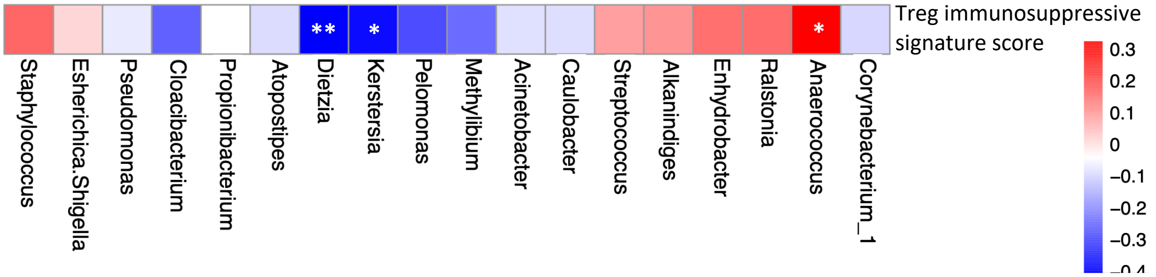

b

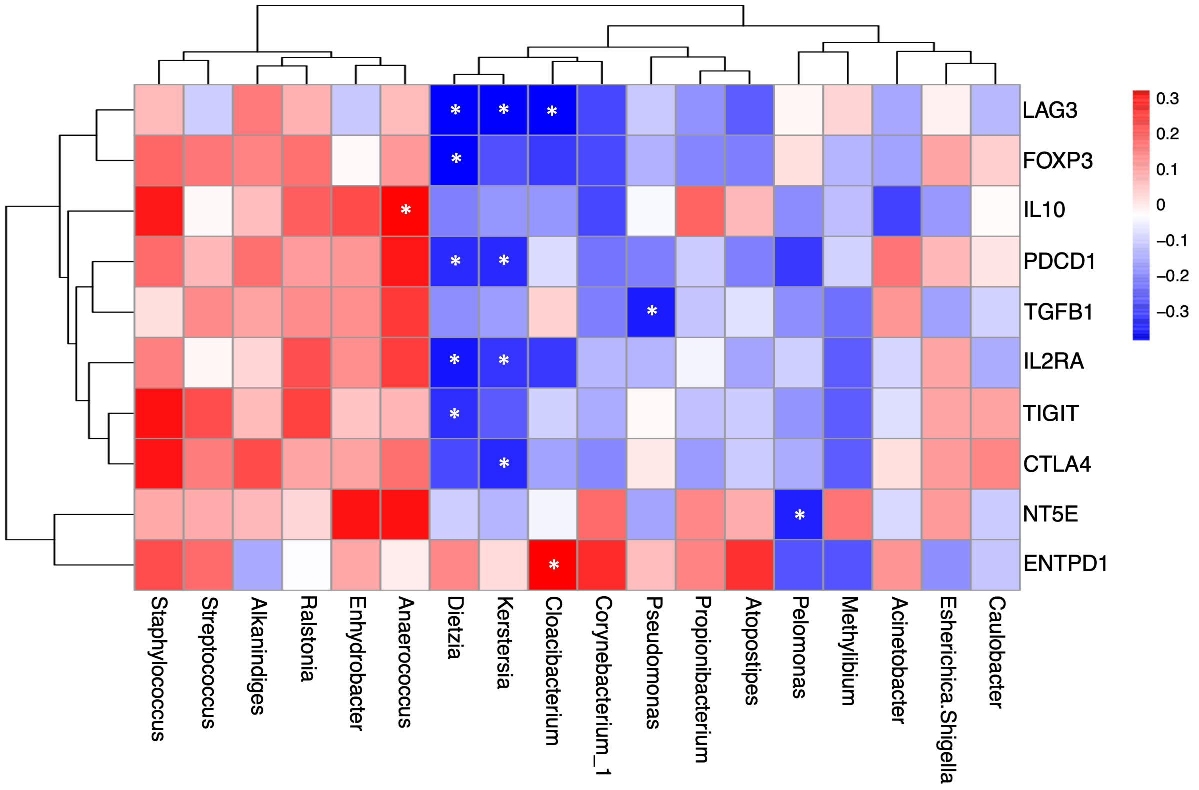

Supplementary figure 2 | Correlations between intratumoral bacterial genera and the Treg-associated immunosuppressive signature. a, b Heatmaps showing Spearman correlations between the abundance of 18 bacterial genera and the composite Treg-associated immunosuppressive signature (**a**) and individual genes comprising the signature (**b**) in Cohort A. *P < 0.05.

Supplementary figure 3

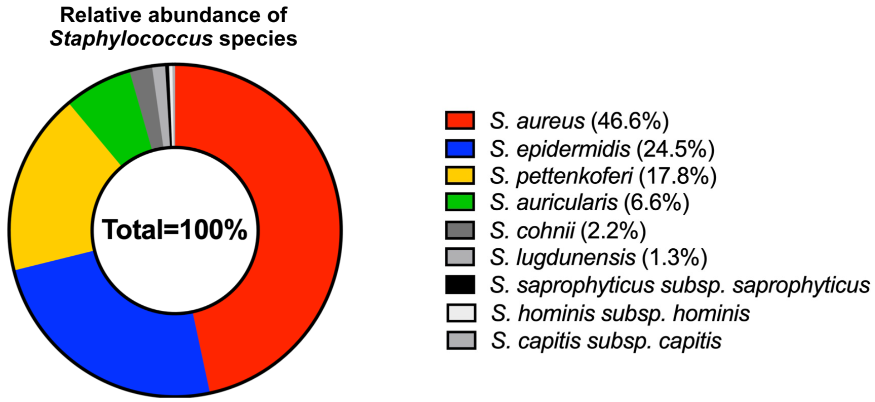

Supplementary figure 3 | The relative abundance of *Staphylococcus* species in human breast tumors. The percentage of *Staphylococcus* species in human breast tumors based on the reads identified in 16S rRNA gene sequencing.

Supplementary figure 4

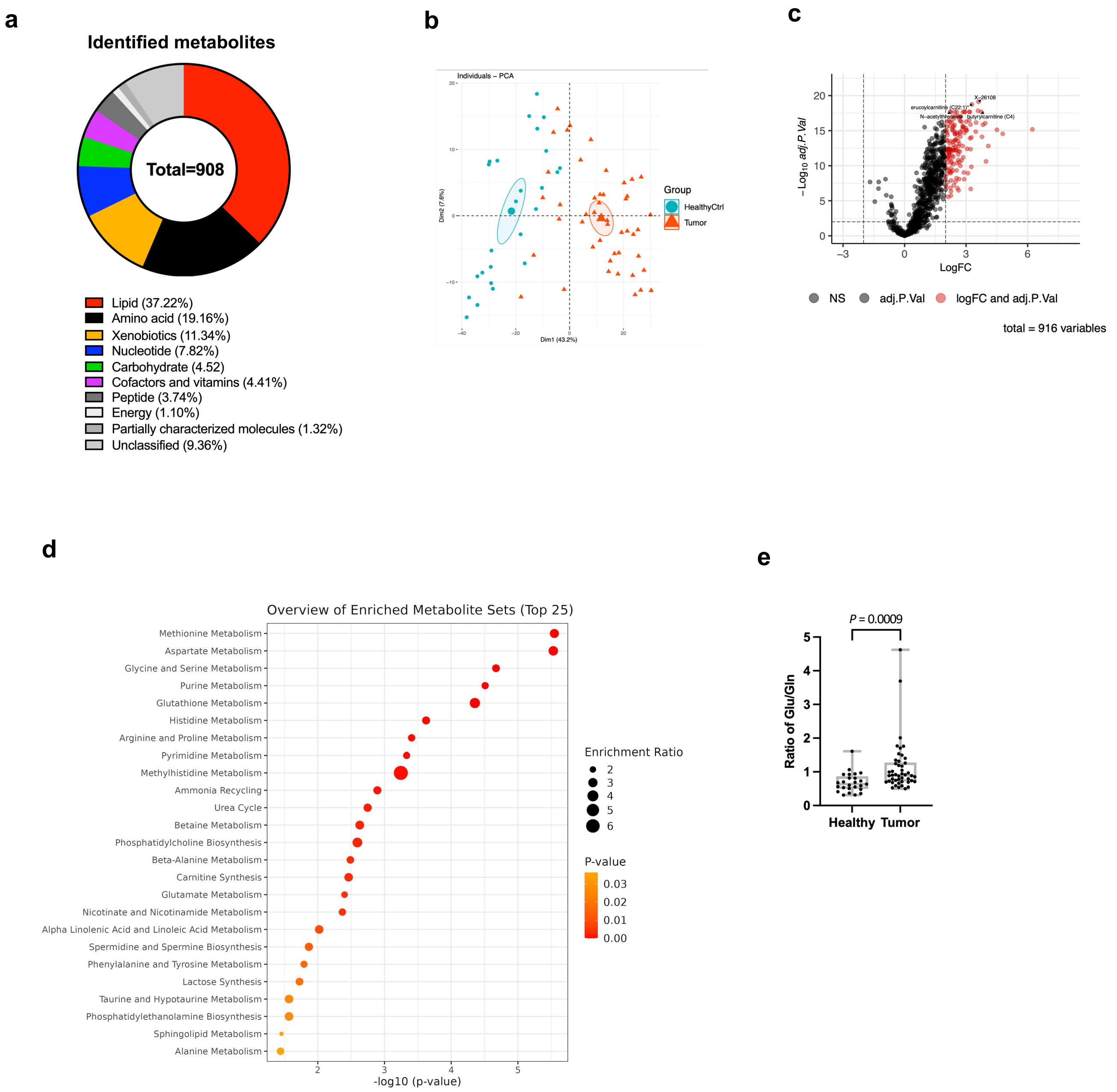

Supplementary figure 4 | Metabolic differences between breast tumors and non-malignant breast tissues. a Composition of the metabolites identified by untargeted metabolomics in human breast tumors and healthy breast tissues. **b** Principal component analysis (PCA) of metabolites in human breast tumors (n=46, in orange) and healthy breast tissues (n=25, in blue). **c** The volcano plot showing the differentially abundant metabolites between breast tumors and healthy breast tissues. Metabolites with a log2 fold change > 2 and –Log10 adjusted p-value > 1.3 are highlighted in red. **d** Top metabolic pathways that are significantly altered in breast tumors compared to healthy breast tissues. **e** Distinct ratios of glutamate (Glu) to glutamine (Gln) between breast tumors and healthy breast tissues.

Supplementary figure 5

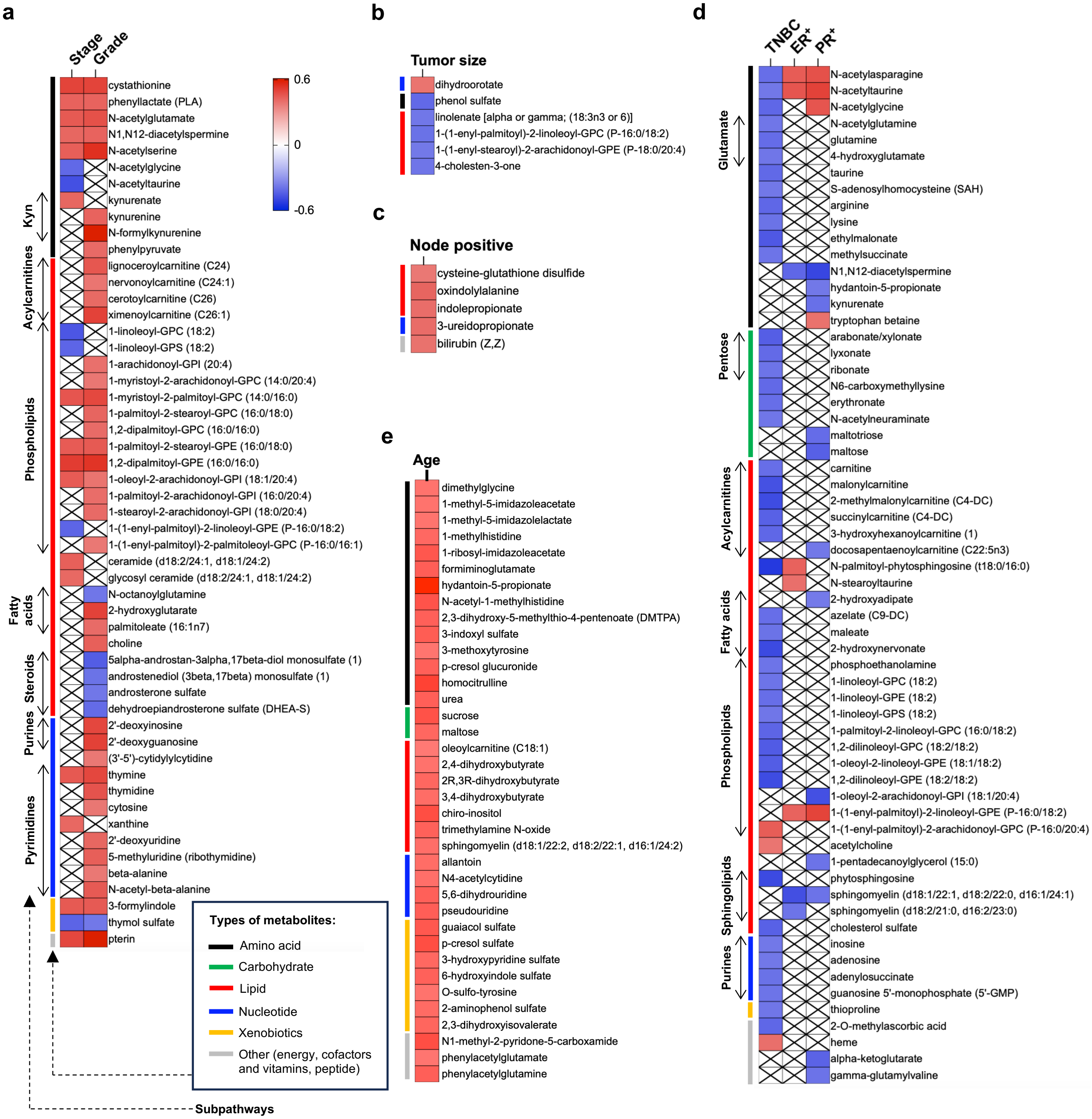

Supplementary figure 5 | Associations between metabolites and clinicopathological features of breast cancer. a-e Heatmaps showing the metabolites significantly associated with cancer stage or histological grade (**a**), tumor size (**b**), lymph node-positive status (**c**), histologic subtypes (**d**), and patient age (**e**) with the color gradient indicating the strength of correlation, and "X" representing non-significant correlations. Types and subpathways of metabolites are indicated on the left side of each heatmap.

Supplementary figure 6

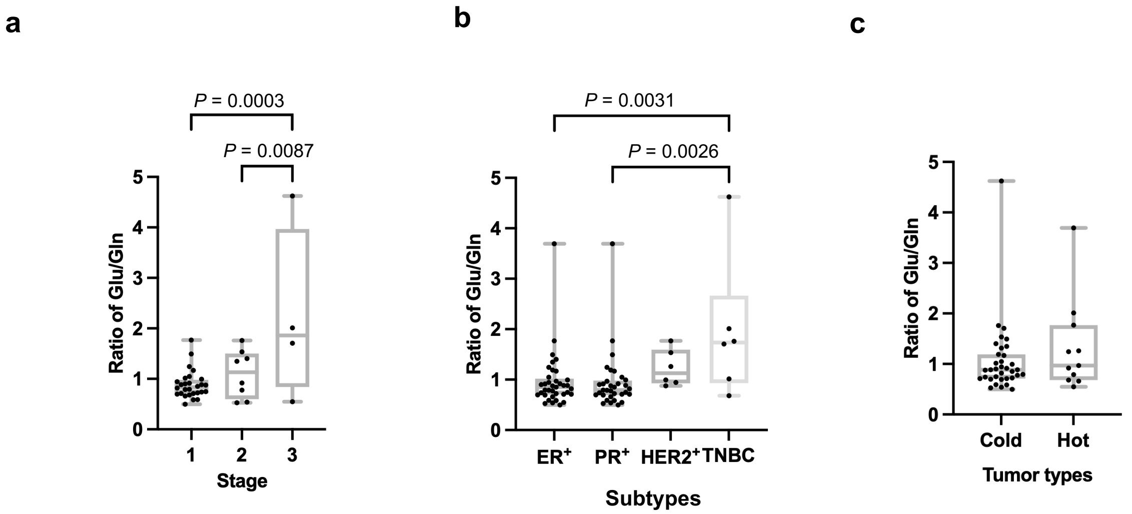

Supplementary figure 6 | Glutamate-to-glutamine ratio across different tumor stages, molecular subtypes, and immune phenotypes. a, b Ratio of glutamate (Glu) to glutamine (Gln) in breast tumors stratified by tumor stages (**a**), molecular subtypes (**b**), and immune phenotypes (**c**; "cold" vs. "hot" defined by low vs. high $CD8^+$ TIL densities). One-way analysis of variance (ANOVA) with multiple comparisons (**a**,**b**) and t-test (**c**) were used, and only statistically significant differences are indicated with p-values.

Supplementary figure 7

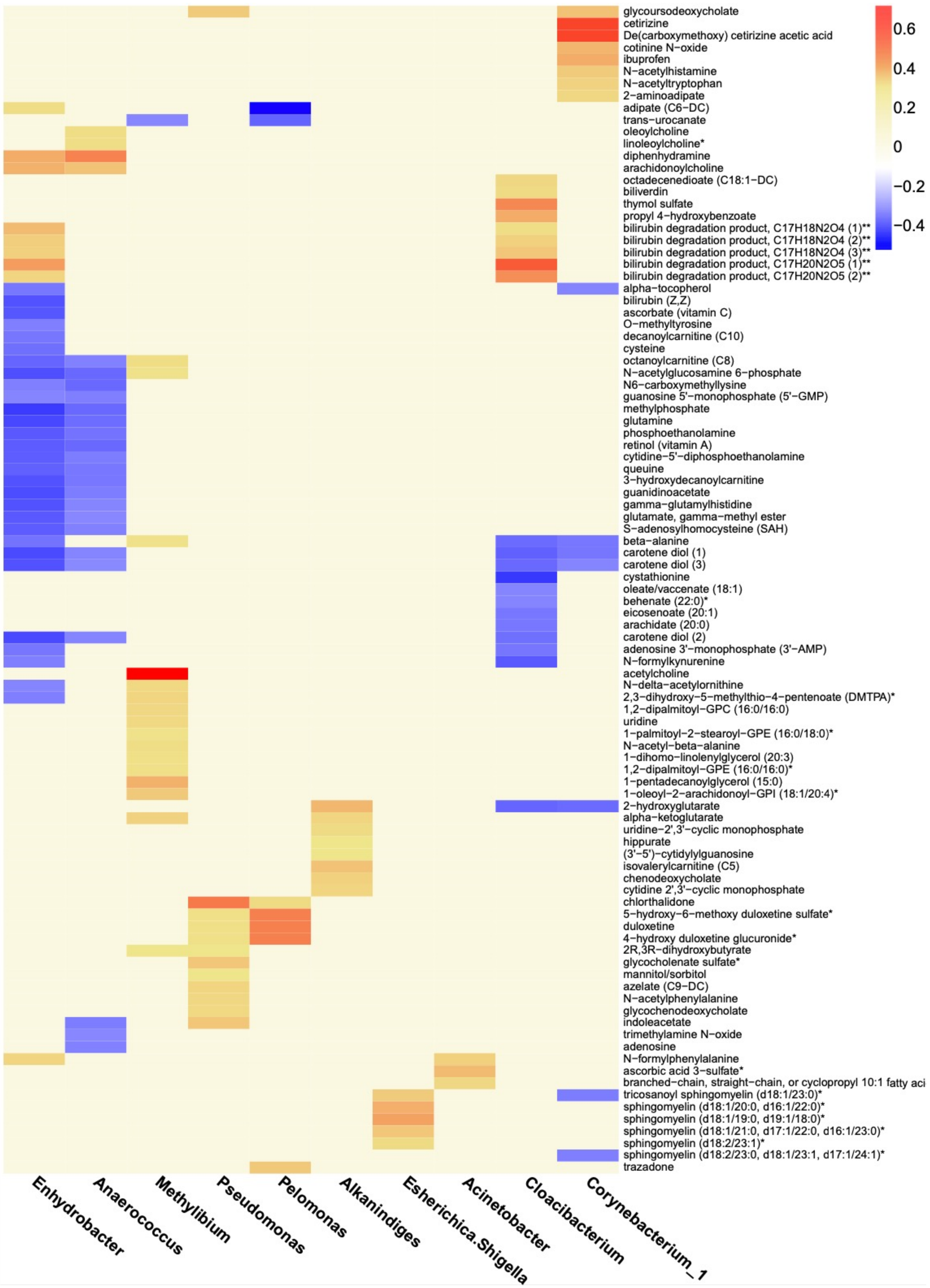

Supplementary figure 7 | Correlations between tumoral bacteria and metabolites. Heatmap displaying significant correlations between the bacterial genera and metabolites within human breast tumors identified by sparse canonical correlation analysis (CCA). The color gradient representing the correlation.

Supplementary figure 8

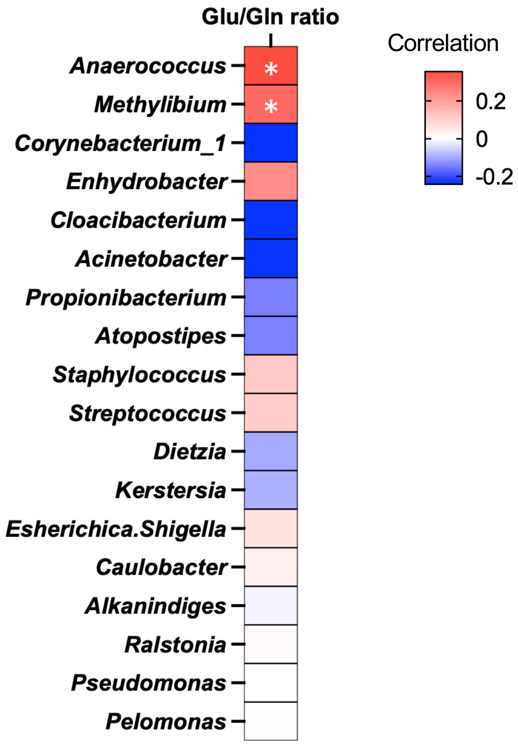

*

Supplementary figure 8 | Correlations between tumoral bacteria and the ratio of glutamate to glutamine. Heatmap illustrating the correlations between bacterial genera and the ratio of glutamate (Glu) to glutamine (Gln). Spearman's rank correlation. *$P < 0.05$.

Supplementary figure 9

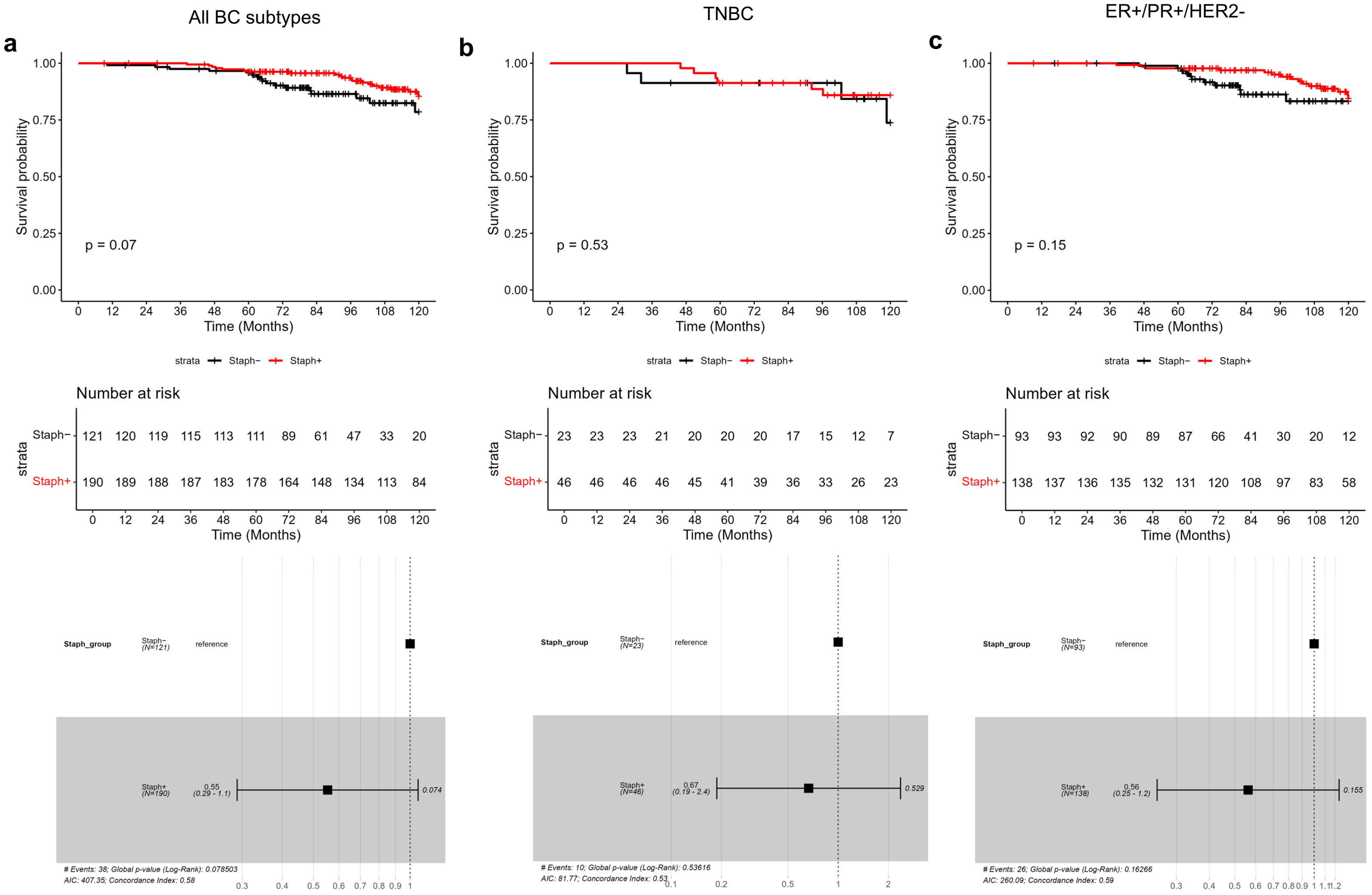

Supplementary figure 9 | Overall survival stratified by intratumoral *Staphylococcus* status across breast cancer subtypes. a-c Kaplan–Meier survival curves illustrating overall survival among patients of all breast cancer subtypes (**a**), TNBC (**b**), and ER+/PR+/HER2- subtype (**c**) stratified by the presence or absence of intratumoral *Staphylococcus* (Staph+ and Staph-, shown as red and black lines, respectively). Tick marks denote censored observations. All BC subtypes: Events: 17/121 (Staph−) vs 21/190 (Staph+). Log-rank p = 0.07. TNBC: Events: 4/23 (Staph−) vs 8/46 (Staph+). Log-rank p = 0.53. ER+/PR+/HER2-: Events: 11/93 (Staph−) vs 15/138 (Staph+). Log-rank p = 0.15. All p-values > 0.05 by log-likelihood test.

Supplementary figure 10

a

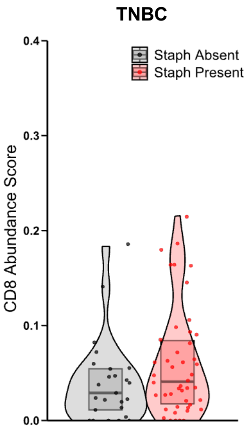

b

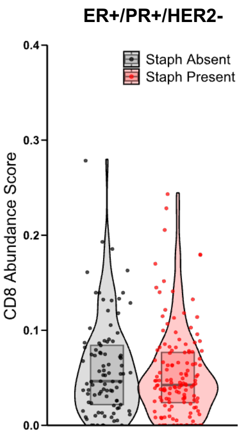

Supplementary figure 10 | Associations between *Staphylococcus* and CD8+ T cell abundance in breast cancer. a, b Comparisons of CD8+ T cell abundance in TNBC (**a**) and ER+/PR+/HER2- tumors (**b**) with and without the presence of *Staphylococcus* (shown as red and black dots, respectively). Z-score transformed signature scores were compared by t-test. All p-values were > 0.05.

Supplementary figure 11

a

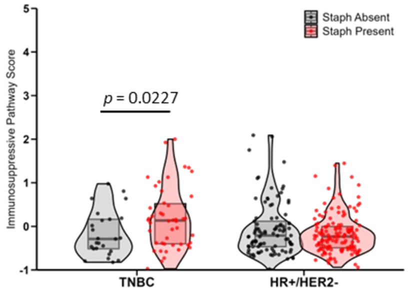

b

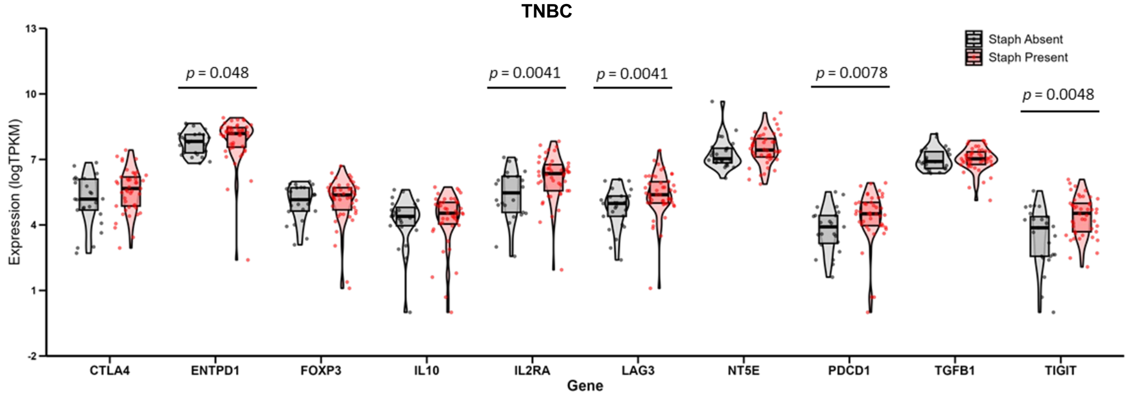

c

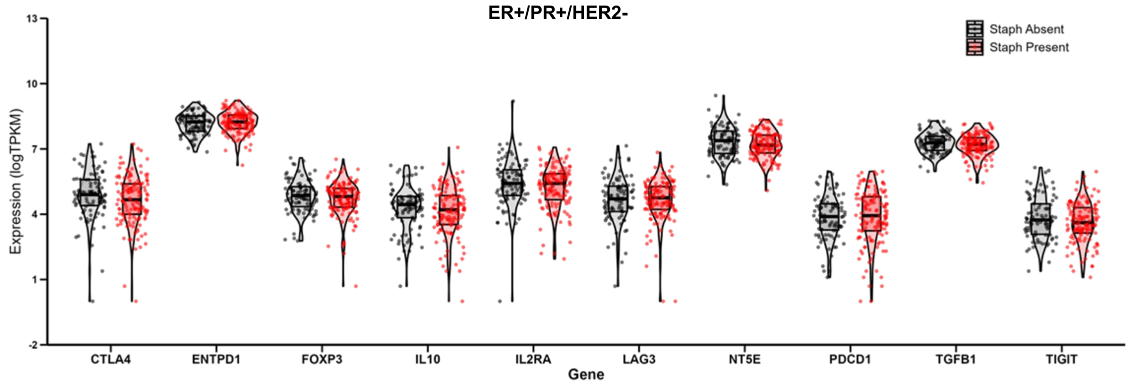

Supplementary figure 11 | Treg-associated immunosuppressive signature in *Staphylococcus*-positive versus *Staphylococcus*-negative breast tumors. a Comparison of the composite Treg-associated immunosuppressive gene signature between *Staphylococcus*-positive and *Staphylococcus*-negative TNBC and ER+/PR+/HER2− tumors in Cohort B. **b, c** Comparisons of individual genes comprising the Treg-associated immunosuppressive signature in *Staphylococcus*-positive versus *Staphylococcus*-negative TNBC (**b**) and ER+/PR+/HER2− tumors (**c**). Only statistically significant differences are shown with p-values.

Supplementary figure 12

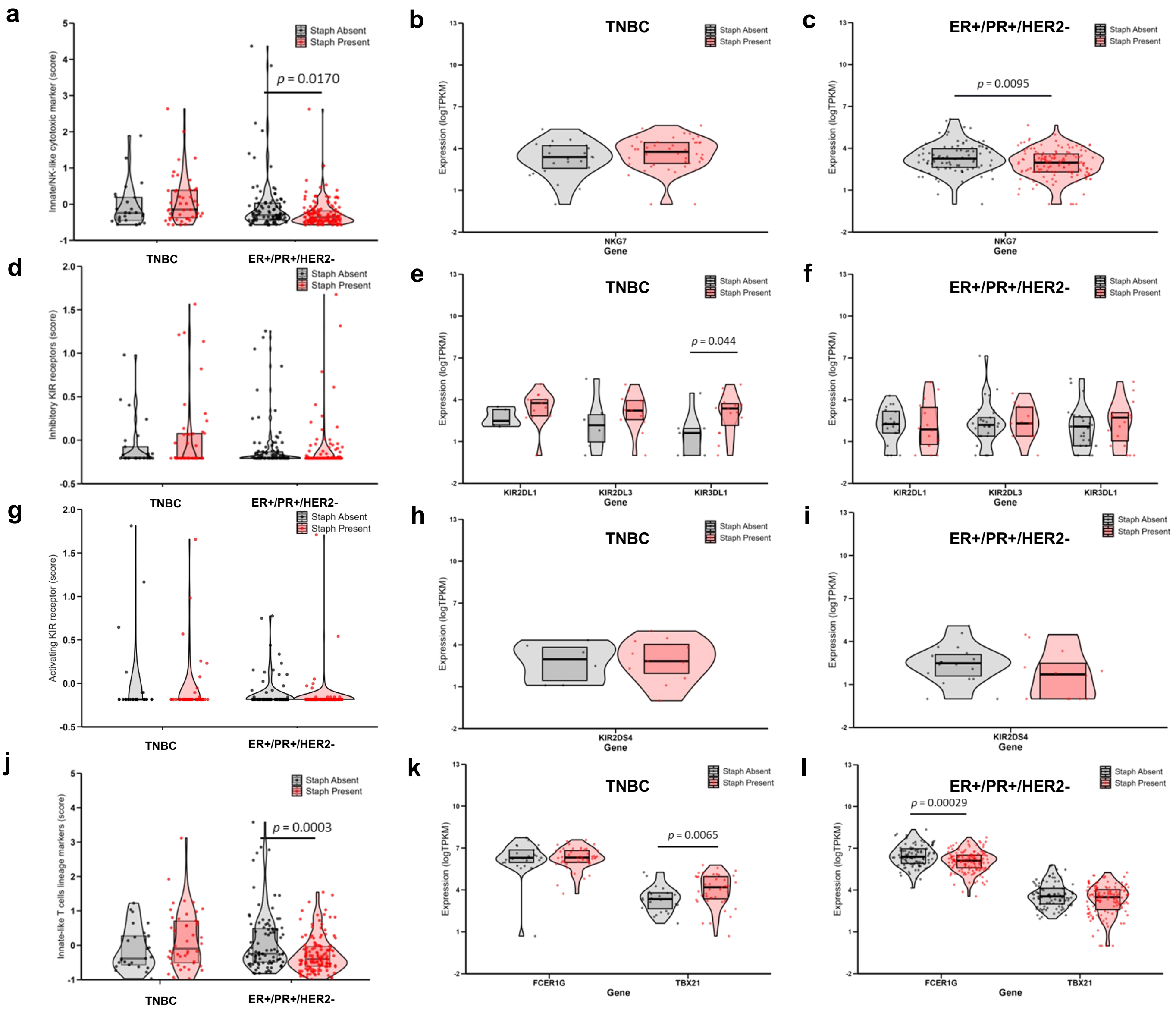

Supplementary Figure 12 | Comparison of innate-like T cell signatures and associated gene expression in *Staphylococcus*-positive versus *Staphylococcus*-negative breast tumors. Signature scores for innate/NK-like cytotoxic markers (**a**), inhibitory KIR receptors (**d**), activating KIR receptors (**g**), and innate-like T cell lineage markers (**j**) were compared between *Staphylococcus*-positive and *Staphylococcus*-negative tumors in TNBC and ER+/PR+/HER2- subtypes. Expression levels of the individual genes comprising each signature are shown in the middle panels (**b, e, h, k**) for TNBC and the right panels (**c, f, i, l**) for ER+/PR+/HER2- tumors. Only statistically significant differences are shown with p-values

Supplementary figure 13

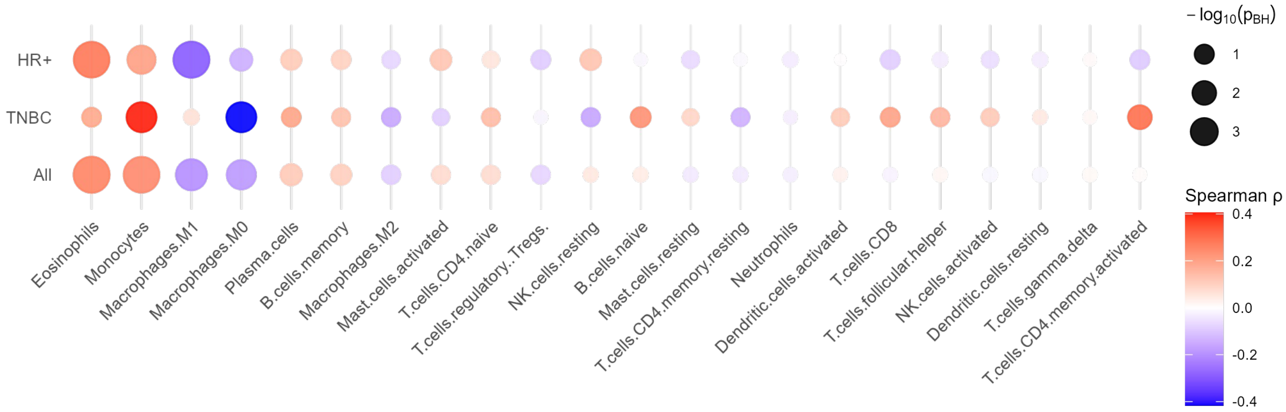

Supplementary figure 13 | Correlations between *Staphylococcus* abundance and immune cell populations across breast cancer subtypes. Spearman correlations between *Staphylococcus* genus count and immune cell abundance in three Cohort B groups: All patients (All), hormone receptor–positive (HR+; ER and/or PR+), and TNBC. Each dot represents one immune cell type. Dot color indicates Spearman's ρ (blue = negative, red = positive; white = 0), and dot size reflects statistical support (–$\log_{10}$ p_{BH} after Benjamini–Hochberg correction). Only paired samples with finite values for both variables were included per test (n indicated in the CSV). Immune-cell columns were coerced to numeric; non-numeric/NA values were excluded pairwise. Immune cells are ordered based on signal in the All cohort (lowest q then |ρ|). See Additional File 1 for the full set of correlations and p-values.

Supplementary figure 14

a

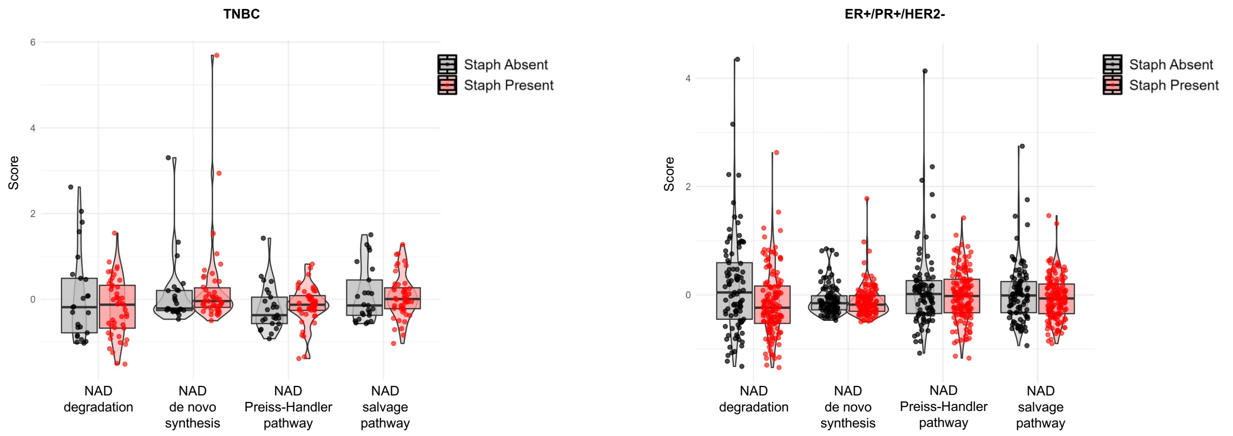

b

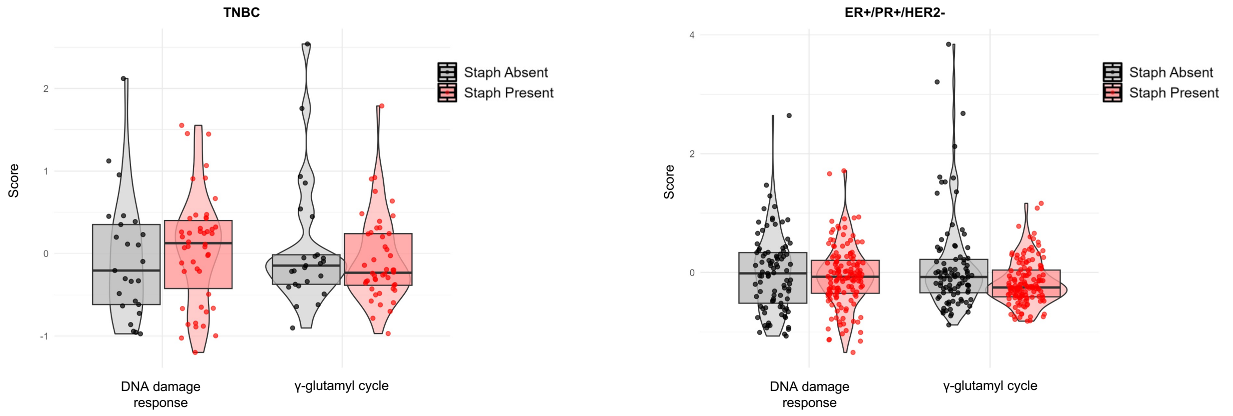

Supplementary figure 14 | Associations between *Staphylococcus* and metabolic pathways in breast cancer. a, b Comparisons of the activity of NAD-related pathways (**a**), DNA damage response (**b**), and γ-glutamyl cycle pathways (**b**) in TNBC (left panels) and ER+/PR+/HER2- subtype (right panels) with and without *Staphylococcus* (Staph, denoted in red and black dots, respectively). Z-score transformed signature scores were compared by t-test. All p-values were > 0.05.

Supplementary figure 15

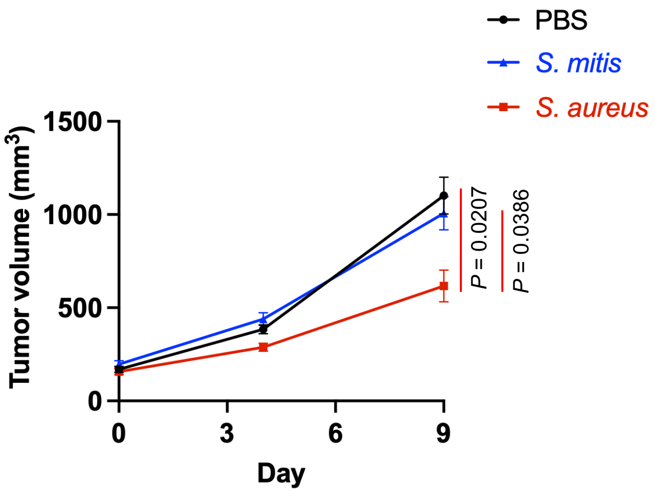

Supplementary figure 15 | Independent biological experiment validating the effects of intratumoral *S. aureus* on EO771 tumor growth. Growth curves of EO771 tumors following intratumoral injection of *S. aureus* (SA) or *S. mitis* (SM), with PBS as control (n=4-5). Data analyzed by two-way analysis of variance (ANOVA) with multiple comparisons.

Supplementary figure 16

a

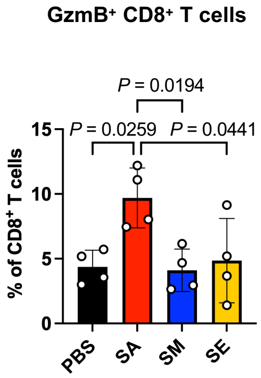

b

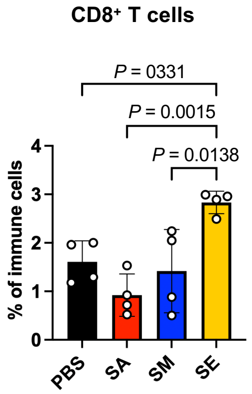

Supplementary figure 16 | Intratumoral *S. aureus* enhance CD8+ T cell activation in 4T1 tumors. a, b 4T1 tumors were colonized by *S. aureus* (SA), *S. mitis* (SM), or *S. epidermidis* (SE), with PBS as control. Flow cytometry was performed to quantify the percentage of GzmB+ CD8+ T cells among CD8+ T cells (**a**) and CD8+ T cells among immune cells (**b**). Statistical analysis was performed using one-way analysis of variance (ANOVA) with multiple comparisons. Only significant differences are indicated with *p*-values.

Supplementary figure 17

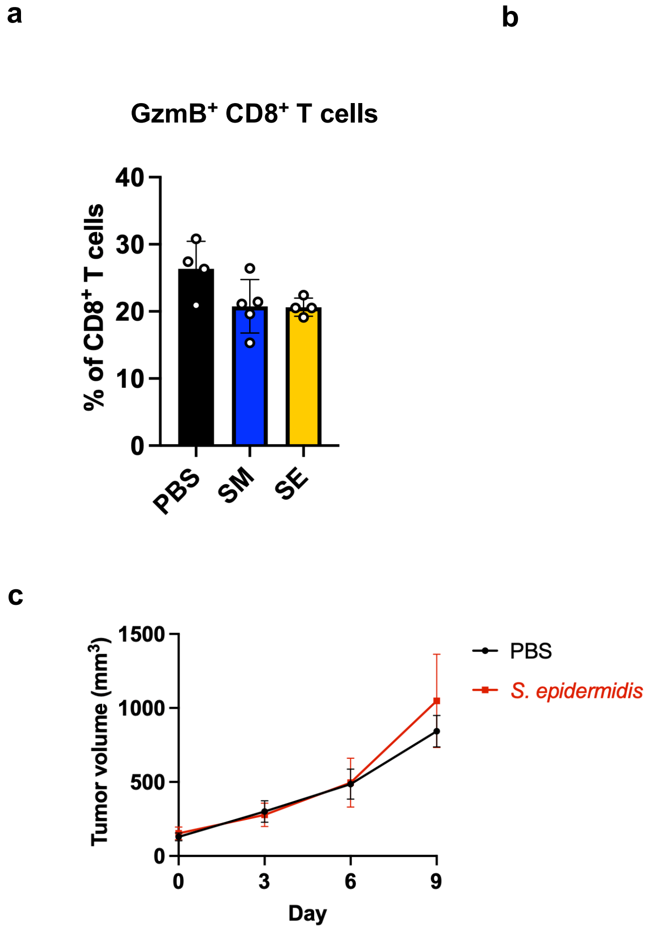

Supplementary figure 17 | Effects of intratumoral *S. epidermidis* on CD8$^+$ T cells and tumor growth in the EO771 model. **a, b** EO771 tumors were injected with *S. mitis* (SM) or *S. epidermidis* (SE), with PBS as a control. Flow cytometry quantified the percentage of GzmB$^+$ CD8$^+$ T cells among CD8$^+$ T cells (**a**) and the proportion of CD8$^+$ T cells among all immune cells (**b**). **c** Growth of EO771 tumors following intratumoral injection of *S. epidermidis* compared to PBS-treated control (n=4). Only significant differences are indicated with *p*-values. One-way analysis of variance (ANOVA) with multiple comparisons (**a, b**). Two-way analysis of variance (ANOVA) with multiple comparisons (**c**). No comparisons reached significance.

Supplementary figure 18

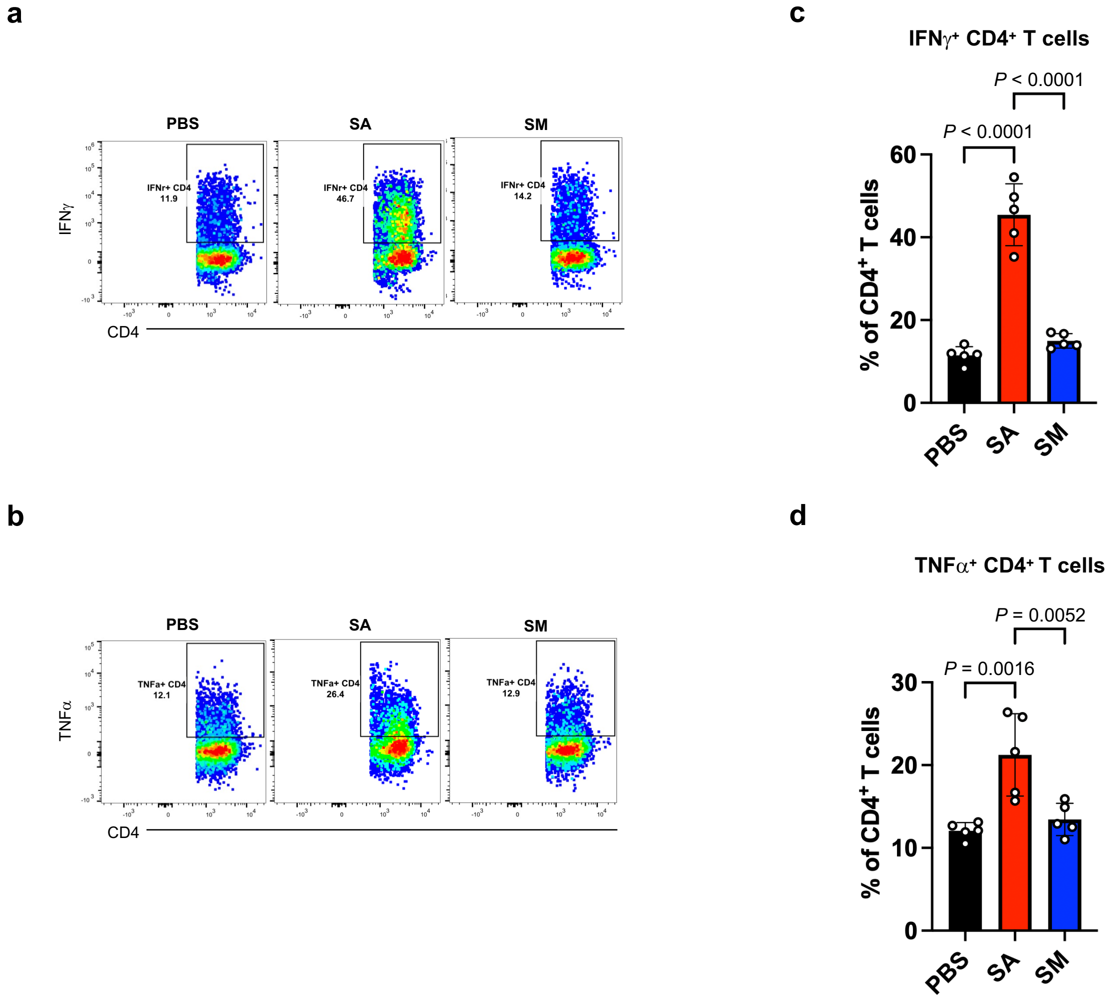

Supplementary figure 18 | Intratumoral *S. aureus* stimulates CD4⁺ T cells in EO771 tumors. a, b Representative flow cytometry dot plots showing IFNγ⁺ (**a**) and TNFα⁺ (**b**) CD4⁺ T cells in EO771 tumors following intratumoral injection with *S. aureus* (SA) or *S. mitis* (SM), with PBS as a control. **c, d** Quantification of IFNγ⁺ (**c**) and TNFα⁺ (**d**) CD4⁺ T cells from the results shown in **a** and **b**. One-way ANOVA with multiple comparisons. Only the significant differences are indicated with *p*-values.

Supplementary figure 19

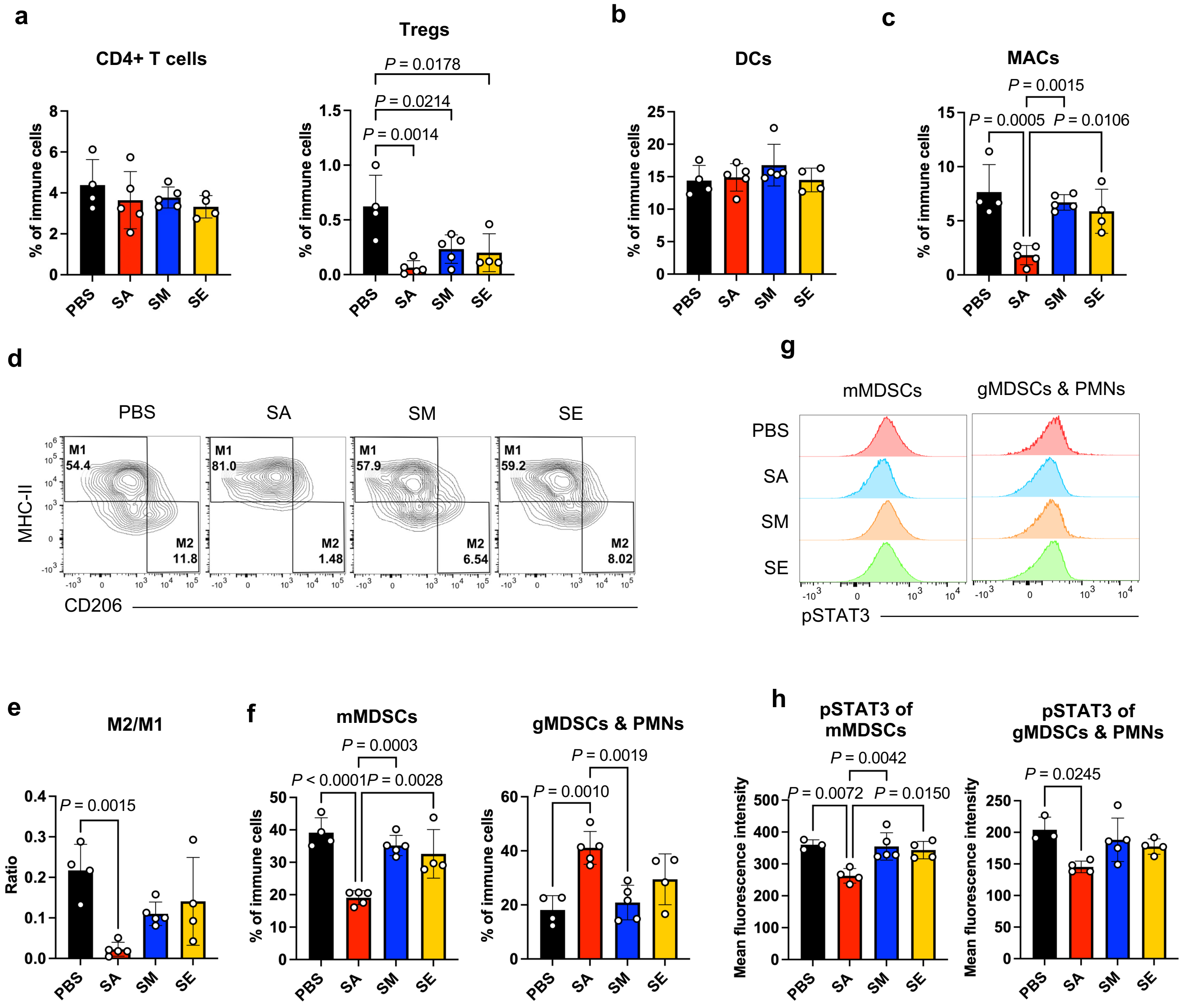

Supplementary figure 19 | Effects of intratumoral *S. aureus* on CD4⁺ T cell and innate immune populations in EO771 tumors. a-c, e, f, h Flow cytometry analysis of EO771 tumors colonized with *S. aureus* (SA), *S. mitis* (SM), or *S. epidermidis* (SE), with PBS as a control. Analyses include: the percentage of CD4+ T cells and Tregs among immune cells (**a**), dendritic cells (DCs) (**b**), macrophages (MACs) (**c**), the M2/M1 MAC ratio (**e**), monocytic myeloid-derived suppressor cells (mMDSCs) and granulocytic MDSC (gMDSCs)/polymorphonuclear neutrophils (PMNs) (**f**), and phosphorylated STAT3 mean fluorescence intensity in mMDSCs and gMDSCs/PMNs (**h**). **d** Representative contour plots showing gating of M2-like (MHC-II^{low}, CD206^{+}) and M1-like (MHC-II^{high}, CD206^{-}) MACs. **g** Representative histograms showing phosphorylated STAT3 levels in mMDSCs and gMDSCs/PMNs. Statistical analysis: one-way analysis of variance (ANOVA) with multiple comparisons (**a**, **b**, **c**, **e**, **f, h**). Only significant differences are indicated with *p*-values.

Supplementary figure 20

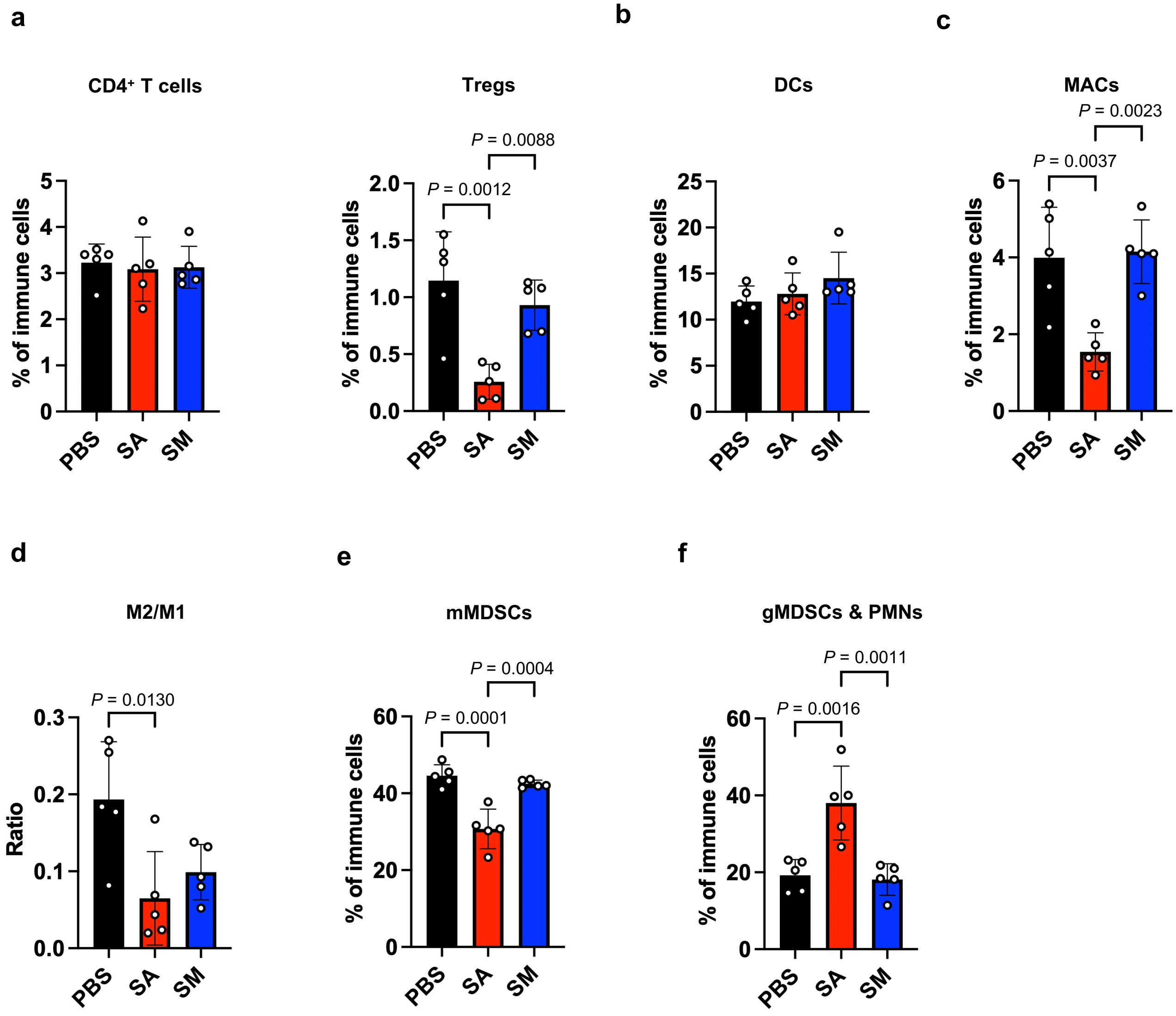

Supplementary Fig. 20 | Independent biological experiment confirming the effects of intratumoral *S. aureus* on CD4⁺ T cell and innate immune populations in EO771 tumors. **a–f** Flow cytometry analysis of EO771 tumors colonized with *S. aureus* (SA) or *S. mitis* (SM), with PBS as a control. Analyses include the percentage of CD4⁺ T cells, Tregs, dendritic cells (DCs), macrophages (MACs), monocytic myeloid-derived suppressor cells (mMDSCs), and granulocytic MDSCs/polymorphonuclear neutrophils (gMDSCs/PMNs) among immune cells (**a–c**, **e–f**), and the M2/M1 MAC ratio (**d**). Statistical analysis: one-way analysis of variance (ANOVA) with multiple comparisons. Only significant differences are indicated with p-values.

Supplementary figure 21

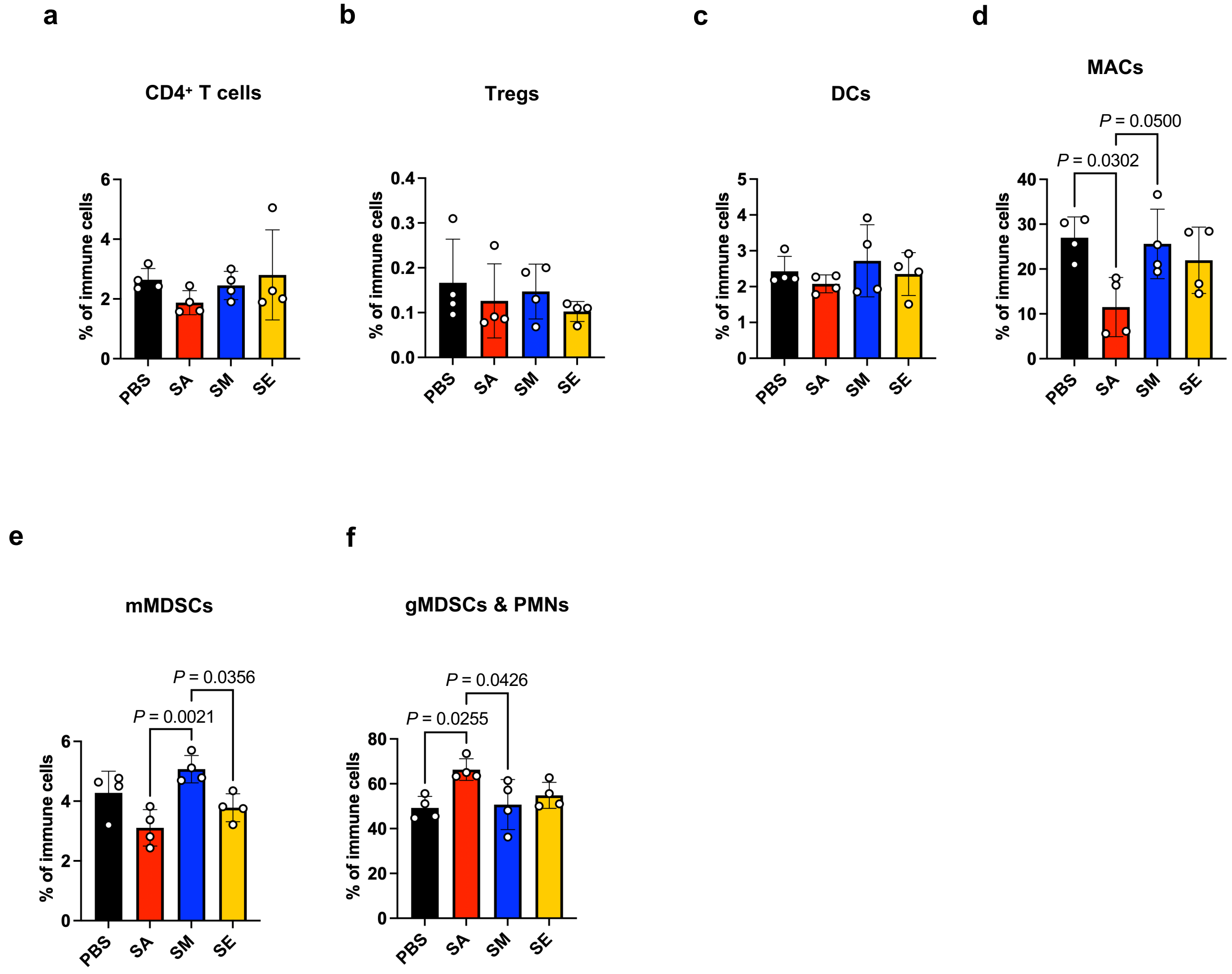

Supplementary figure 21 | Effects of intratumoral *S. aureus* on CD4^{+} T cells and innate immune populations in 4T1 tumors. a-f Flow cytometry analysis of 4T1 tumors colonized with *S. aureus* (SA), *S. mitis* (SM), or *S. epidermidis* (SE), with PBS as a control. Analyses include the percentages of CD4^{+} T cells, Tregs, dendritic cells (DCs), macrophages (MACs), monocytic myeloid-derived suppressor cells (mMDSCs), and granulocytic MDSCs/polymorphonuclear neutrophils (gMDSCs/PMNs) among immune cells. Statistical analysis: one-way analysis of variance (ANOVA) with multiple comparisons. Only significant differences are indicated with *p*-values.

Supplementary figure 22

a

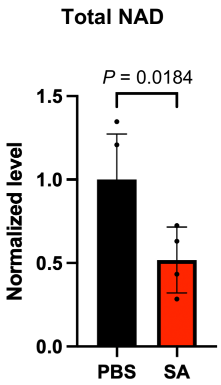

Supplementary figure 22 | Independent biological experiment confirming the effects of intratumoral *S. aureus* on total NAD levels in EO771 tumors. Total levels of NAD+ and NADH in the EO771 tumors six days after intratumoral injection of *S. aureus* (SA), with PBS treatment as a control. Unpaired two-tailed Student's t-test.

Supplementary figure 23

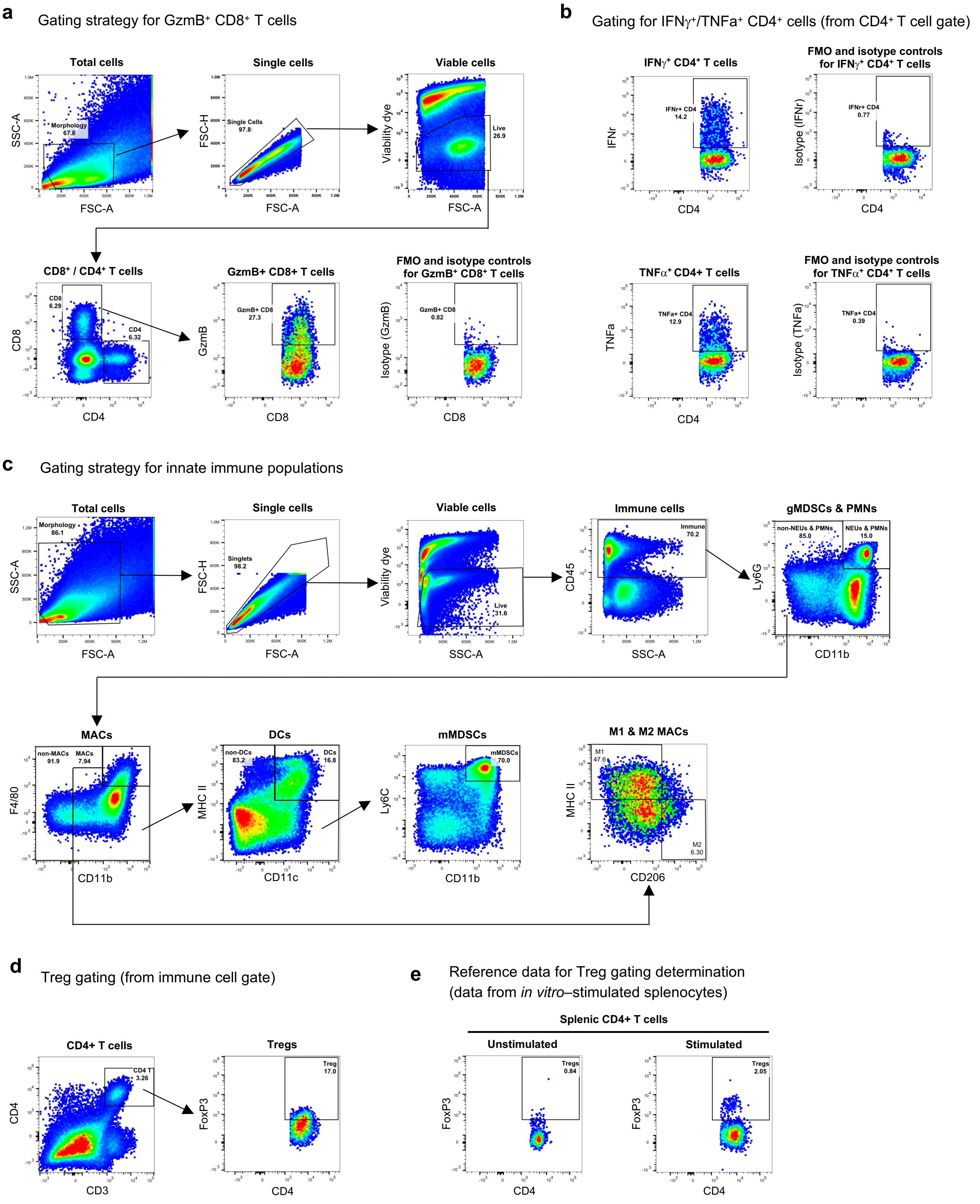

Supplementary figure 23

Supplementary Figure 23 | Flow cytometric gating strategies used throughout the study. a Identification of GzmB$^+$ CD8$^+$ T cells, including FMO and isotype controls for GzmB staining. Events recorded per gate: total cells, 458,230; single cells, 448,047; viable cells, 120,674; CD8$^+$ T cells, 7,590; CD4$^+$ T cells, 7,623; and GzmB$^+$ CD8$^+$ T cells, 2,074. **b** Identification of IFNγ$^+$ and TNFα$^+$ CD4$^+$ T cells, with FMO and isotype controls for IFNγ and TNFα staining. Events per gate: CD4$^+$ T cells, 7,623; IFNγ$^+$ CD4$^+$ T cells, 1,083; TNFα$^+$ CD4$^+$ T cells, 985. **c** Identification of innate immune populations. Events per gate: total cells, 845,965; single cells, 831,003; viable cells, 262,827; immune cells, 184,535; granulocytic myeloid-derived suppressor cells (gMDSCs) and polymorphonuclear leukocytes (PMNs), 27,640; macrophages (MACs), 12,464; M1-like MACs, 5,936; M2-like MACs, 785; dendritic cells (DCs), 24,341; and monocytic MDSCs (mMDSCs), 83,992. **d** Identification of Tregs. Events per gate: immune cells, 184,535; CD4$^+$ T cells, 6,018; Tregs, 1,026. **e** In vitro splenic T-cell stimulation used as reference to guide Treg (CD4$^+$FOXP3$^+$) gating in tumors. Events per gate: left figure (unstimulated), 22; right figure (stimulated), 149.

Supplementary Table 1 | The top 25 metabolites significantly enriched in breast tumors compared to healthy breast tissues.

Metabolites enriched in control breast tissues	Log2 fold change (tumor/control)	Adjusted p-value
erucoylcarnitine (C22:1)*	3.28	1.75E-19
arachidoylcarnitine (C20)*	3.58	1.45E-18
nervonoylcarnitine (C24:1)*	3.16	2.11E-18
docosadienoylcarnitine (C22:2)*	3.18	2.11E-18
butyrylcarnitine (C4)	3.74	2.11E-18
behenoylcarnitine (C22)*	3.15	3.40E-17
glycerophosphoethanolamine	3.01	5.36E-17
glutamate, gamma-methyl ester	3.49	6.99E-17
5-methylthioadenosine (MTA)	3.93	8.58E-17
eicosenoylcarnitine (C20:1)*	3.22	1.35E-16
N-acetylaspartate (NAA)	3.82	1.45E-16
ascorbate (vitamin C)	6.23	6.55E-16
phytosphingosine	4.50	7.50E-16
N-acetylputrescine	3.00	8.86E-16
N-palmitoyl-phytosphingosine (t18:0/16:0)	3.47	1.39E-15
alpha-tocopherol	4.79	2.80E-15
cytidine 5'-diphosphocholine	3.78	7.57E-15
quinolinate	3.26	9.18E-15
ethylmalonate	3.19	1.23E-14
UDP-N-acetylglucosamine/galactosamine	4.08	1.52E-13
N-acetyl-aspartyl-glutamate (NAAG)	3.05	3.85E-13
cystathionine	3.97	2.43E-11
guanosine 5'-monophosphate (5'-GMP)	3.26	1.44E-10
uridine 5'-monophosphate (UMP)	3.09	1.15E-09
glutathione, reduced (GSH)	3.23	2.10E-07

Mann-Whitney U test

Supplementary Table 2 | The top 25 metabolites significantly enriched in healthy breast tissues compared to breast tumors.

Metabolites enriched in control breast tissues	Log2 fold change (tumor/control)	Adjusted p-value
sphingomyelin (d18:1/20:1, d18:2/20:0)*	-0.93	8.33E-09
trans-urocanate	-1.68	2.07E-08
linolenate [alpha or gamma; (18:3n3 or 6)]	-0.89	1.64E-06
sphingomyelin (d18:1/18:1, d18:2/18:0)	-0.65	2.71E-06
sphingomyelin (d18:2/24:2)*	-0.65	3.07E-05
3-hydroxy-2-methylpyridine sulfate	-0.63	5.93E-05
caprylate (8:0)	-0.60	0.00011136
bilirubin degradation product, C17H20N2O5 (2)**	-0.70	0.00030644
octadecadienedioate (C18:2-DC)*	-0.79	0.00051615
branched-chain, straight-chain, or cyclopropyl 10:1 fatty acid (1)*	-0.61	0.00087754
triethanolamine	-0.63	0.00105426
o-cresol sulfate	-0.65	0.00128545
13-HODE + 9-HODE	-0.47	0.00345925
1-(1-enyl-palmitoyl)-2-linoleoyl-GPC (P-16:0/18:2)*	-0.58	0.00820577
androstenediol (3beta,17beta) disulfate (1)	-0.79	0.00902972
3-amino-2-piperidone	-0.64	0.00986265
octadecenedioate (C18:1-DC)	-0.54	0.01034876
sphingomyelin (d18:2/18:1)*	-0.52	0.01066854
4-vinylphenol sulfate	-0.79	0.0109589
N-behenoyl-sphingadienine (d18:2/22:0)*	-0.39	0.01771484
12,13-DiHOME	-0.54	0.0190684
5alpha-androstan-3beta,17beta-diol disulfate	-0.66	0.02234858
fructosyllysine	-0.76	0.02345034
chenodeoxycholate	-0.56	0.03225341
2-aminophenol sulfate	-0.48	0.04465686

Mann-Whitney U test
